# Second Harmonic Nonlinear Warburg Admittance Analysis Eliminates Information Loss from Linearization in Traditional EIS: Theory and Experimental Validation

**DOI:** 10.1021/acs.analchem.6c01245

**Published:** 2026-08-05

**Authors:** Lauren A. Frank, Jerome T. Babauta, Daniel T. Schwartz

**Affiliations:** † Department of Chemical Engineering and Clean Energy Institute, 7284 University of Washington, Box 351750, Seattle, Washington 98195-1750, United States; ‡ 440052 Gamry Instruments, Inc., 734 Louis Drive, Warminster, Pennsylvania 18974, United States

## Abstract

Low-frequency impedance or admittance experiments of reversible redox couples in well-supported quiescent electrolyte are normally analyzed using a linearized semi-infinite Warburg element with a single lumped parameter. Because each species of a redox couple has a distinct diffusivity, one cannot determine individual diffusivities by fitting a spectrum with one parameter; linearization causes information loss. Weakly nonlinear theory and experiments presented here extend traditional Warburg admittance analysis to the higher harmonic currents generated from single-sine potential modulations at a Pt electrode in well-supported ferri-/ferro-cyanide electrolyte. Complex harmonic current data from our commercial instrument is processed to enable direct fitting of theory to measurements. We show that complex first and second harmonic currents are measurable for Δ*E* ≥ 5 mV_*rms*_, whereas a good compromise between experimental signal quality and leading-order model accuracy is found at Δ*E* ≈ 10 mV_*rms*_. Higher-order corrections to theory are required at larger potential modulation amplitudes to remove amplitude dependency from fits, until Δ*E* × $\frac{F}{\mathrm{RT}}$ > 1, when weakly nonlinear theory breaks down. Fitting the first and second harmonic experimental spectra to theory provides the mean diffusivity and a diffusion asymmetry parameter (deviations from mean), respectively, enabling unique determination of the individual ferricyanide and ferrocyanide diffusivities. Theory also shows that even harmonics disappear in the high-symmetry case of equal species diffusivities. In short, analyzing the first and second harmonics generated with moderate amplitude modulations eliminates information loss from linear Warburg analysis of the first harmonic alone.

## Graphical abstract

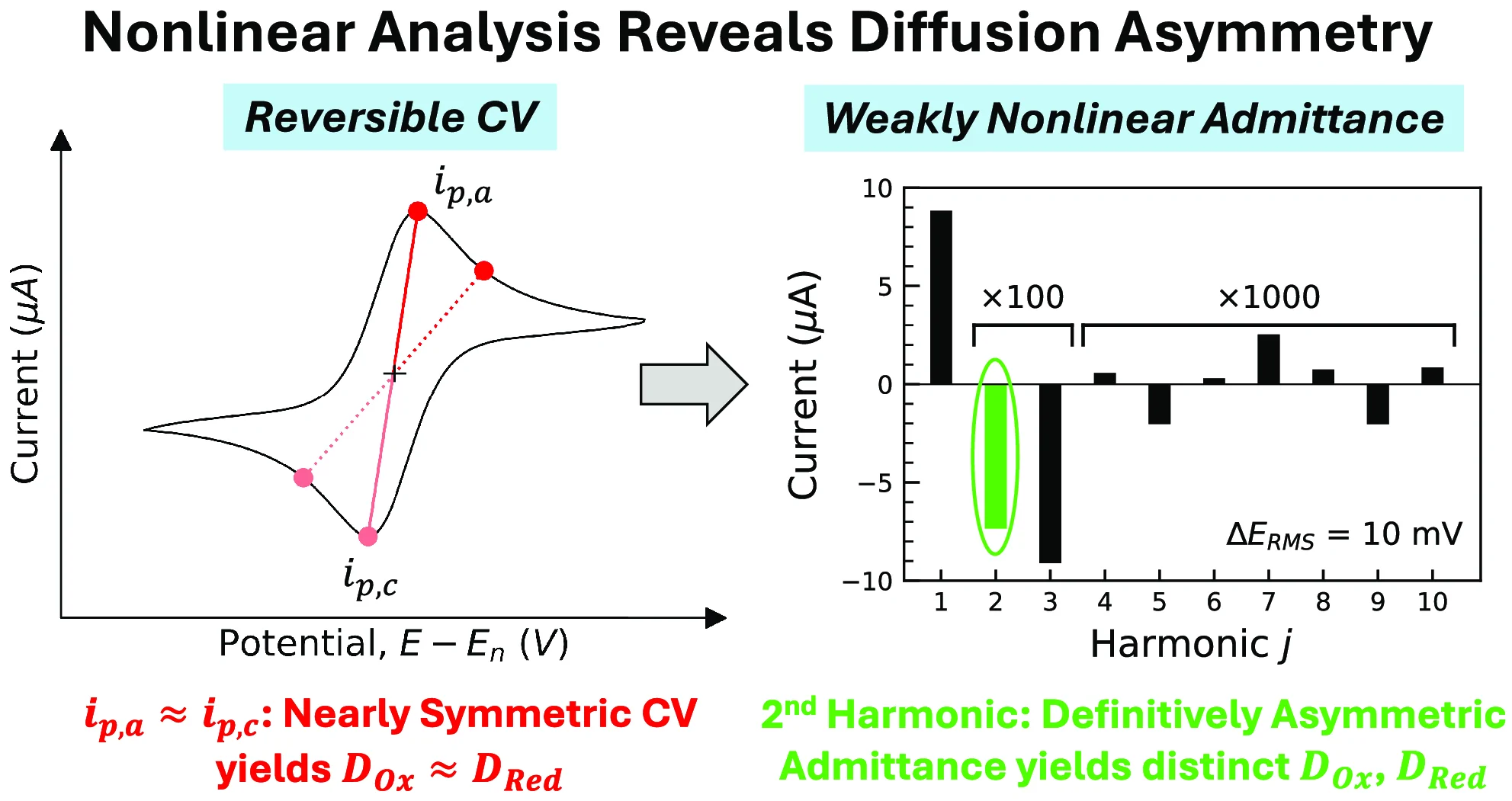

## Introduction

Perhaps the most common model system in electroanalytical chemistry is a dilute, reversible redox couple in a large volume of well-supported quiescent electrolyte. For this system, electrode potential and surface concentrations are related through Nernstian thermodynamics while charge transfer rates are limited by Fickian diffusion. In a cyclic voltammogram (CV), this system defines “reversible peak splitting”, while the square-root dependence of peak heights on scan rate can be used to determine each redox species’ diffusivity (via the Randles–Sevcik equation).^1^ Consequently, CVs are routinely used to classify the reversibility of a wide range of redox systems, but careful quantitation of species diffusivities can be fraught owing to poor faradaic signal-to-capacitive background (especially for low solubility biomolecules).^2–4^ Moreover, even a simple redox couple like ferri-/ferro-cyanide has sufficiently subtle electronic, structural, and hydration shell changes^5,6^ that alternatives to CVs are commonly used for careful quantitation of individual species diffusivities.^7–11^

The semi-infinite Warburg impedance (or admittance) function is based on linearized analysis of the same Nernstian/Fickian model electrochemical system. Impedance or admittance experiments probe near-equilibrium behavior using small amplitude current or potential modulations, respectively, of varying frequency, and can readily separate capacitive and faradaic processes in the output response.^12^ Unfortunately, small amplitude linearization inherent to Warburg measurements reduce the information content compared to a CV; only the averaged diffusivity for the reduced and oxidized species can be determined from a single EIS spectrum, not the individual diffusivities as with a CV.^1,13,14^

The information gap between linearized measurements and fully nonlinear dynamic experiments (such as a typical CV) can often be bridged through weakly nonlinear impedance theory, without requiring any change to EIS experimental procedures.^15–21^ In particular, second harmonic nonlinear EIS (2nd-NLEIS) is sensitive to physicochemical processes that break the inversion symmetry of electrochemical current–potential relationships.^16,17,22,23^ Inversion symmetry in electrochemical processes is common; the CV for an equimolar reversible redox couple has inversion symmetry through the stationary equilibrium potential if the reduced and oxidized species have equal diffusivities. This inversion symmetry is broken when the diffusivities deviate from each other. Because 2nd-NLEIS measurements are sensitive to inversion-symmetry-breaking, we hypothesize simultaneous measurements of EIS and 2nd-NLEIS will have the experimental benefits of EIS (separating faradaic and nonfaradaic currents) while enabling independent determination of both species’ diffusivities in a single experiment.

We were encouraged to develop weakly nonlinear Warburg functions based on two recently published works. First, Goh et al. performed potentiostatic EIS (PEIS) experiments using dilute, well-supported, equimolar ferri-/ferro-cyanide electrolyte to quantify nonlinear distortion to the linear Warburg element as root-mean-squared (rms) potential modulation amplitude was varied over the range 1 mV_*rms*_ to 100 mV_*rms*_ (around the stationary Nernst potential).^24^ The ferri-/ferro-cyanide electrolyte and platinum working electrode used by Goh et al. are classic representations of a reversible model system. Goh et al. showed that single-sine EIS experiments with potential modulations ≥5 mV_*rms*_ had total harmonic distortion (THD) spectra possessing readily measurable second and higher-order harmonics. The spectra were considered to have acceptable linearity for conventional EIS analysis with potential modulations ≤10 mV_*rms*_, where THD was ≤1.1%. In contrast, signals generated by potential modulations ≥20 mV_*rms*_ and THD > 4% were deemed to be excessively influenced by nonlinearity and not well suited for leading-order linear Warburg analysis. Thus, in the modulation window 5 mV_*rms*_ ≤ Δ*E* ≤ 10 mV_*rms*_ linearity was deemed suitable for conventional analysis at the same time nonlinear harmonics were readily measurable. In this paper, we develop theory for analyzing the second harmonic signal from these experiments, then fit experiments to theory to show, for the first time, how to use the second harmonic to extract individual diffusivities in redox couples that possess small diffusivity differences, where CV-based quantitation can be challenging.

The second motivating publication is our recent release of the open-source software toolbox *nleis.py* for analysis of 2nd-NLEIS signals.^22,23^ Our prior work has developed several 2nd-NLEIS finite-length diffusion impedance elements appropriate for batteries, but we have not included a semi-infinite Warburg element for 2nd-NLEIS analysis of more conventional electroanalytical configurations. Because the analysis presented here is for the second harmonic frequency dispersion function driven by potential modulations rather than current modulation, we are more properly analyzing an electrochemical “admittance” experiment. For linear systems, the reciprocal relationship between admittance and impedance makes this a trivial distinction, hence the common use of PEIS and galvanostatic EIS (GEIS) designations. However, in nonlinear harmonic generation, differences in the governing equation structure and constraints for potential vs current modulation make reciprocal relations for the second harmonic impedance and admittance nontrivial.^25^ Thus, we present all experimental data and analysis in its natural admittance form, as measured, and show that conventional EIS or admittance experiments deemed suitable for linear analysis can contain useable harmonic data for simultaneous analysis, thereby eliminating information loss associated with linearization.

## Experimental Section

### Electrochemical System and Measurements

Equimolar ferri- and ferro-cyanide electrolyte (1.923 mM) was made by mixing 20 mL of 50 mM KCl with 800 μL of 50 mM ferri-/ferro-cyanide stock solution. For measurements, a 3 mm diameter (7.1 mm^2^) platinum disk electrode embedded in a PEEK shroud was submerged into the solution. The working electrode was polished with 0.05 μm alumina slurry, rinsed with DI water, and cleaned in a sonicating bath to minimize contamination and fouling effects. A platinum wire was used as a pseudoreference electrode to prevent drift associated with filling solution leakage and a graphite rod as a counter electrode. The experiment was conducted at room temperature, approximately 298 K.

A single-sine input potential was applied using a Gamry Interface 1010E potentiostat with root-mean-squared modulation amplitudes of 1 mV_*rms*_, 5 mV_*rms*_, 10 mV_*rms*_, 20 mV_*rms*_, 50 mV_*rms*_, and 100 mV_*rms*_. To align with theory, these RMS potentials were converted to peak amplitudes by multiplying each value by √2, yielding modulations of 1.414 mV, 7.071 mV, 14.142 mV, 28.284 mV, 70.711 mV, and 141.421 mV, respectively. At each modulation amplitude, frequency was swept from approximately 0.095 Hz to 1.5 MHz, measured in order of smallest to largest amplitudes to reduce fouling effects from larger overpotentials. The real and imaginary components of the output current were measured for harmonics *j* from *j* = 1 to *j* = 10 by enabling harmonic analysis via the total harmonic distortion (THD) software setting. Enabling THD turns off hardware filters to allow harmonic measurement up to *j* = 10. It was shown to have little benefit in data stabilization.^24^ For the Warburg analysis, the 12 lowest frequencies were used, from 0.095 Hz to 1.196 Hz, to reduce the influence of interfacial capacitance or charge transfer kinetics on the diffusion-limited rates. This choice is mostly arbitrary, retaining enough low-frequency points to reduce signal-to-noise effects near zero frequency while excluding higher-frequency points outside the diffusion-dominated regime. See Figures S4 and S5 in the Supporting Information for further exploration of this 12-point cutoff, and Figure S3 to see the influence of high-frequency effects on the diffusion-limited theory.^26^

### Experimental Data Processing

The potentiostat output data was downloaded in .csv format with column names of {*Freq, IHr1, IHi1, IHr2, IHi2, ... IHr10, IHi10, VHr1, VHi1,VHr2, VHi2, ... VHr10, VHi10*}, where *Freq* is the input modulation frequency (Hz), *IHrj* and *IHij* are the real and imaginary currents (A), and *VHrj* and *VHij* are the real and imaginary voltages (V), for a given harmonic *j*. Harmonic currents and voltages were corrected by a factor of 2/*N* to account for instrument scaling and to report them as the peak amplitudes used in theory, where *N* is the number of samples (*N* = 128). Note that parameter fitting of each model used the angular frequency, ω (rad/s), not the linear frequency (Hz).

All experimental current harmonic data were rotated to represent the response from a cosine potential modulation of amplitude Δ*E* using the phase angle (θ) determined from the experimental first harmonic potential

1 θ=−arctan2(VHi1,VHr1)

where *VHr1* and *VHi1* are the real and imaginary first harmonic voltages from the instrument provided .csv data. To rotate each complex current by the voltage phase angle, a rotation matrix multiplied each current harmonic according to

2 [Re{Ĩj,rot}Im{Ĩj,rot}]=[cos⁡jθ−sin⁡jθsin⁡jθcos⁡jθ]×[IHrjIHij]

where *j* is the integer harmonic number. This phase rotation operation ensures alignment with a purely real input potential, as used in the theoretical analysis for higher harmonic response (below), while preserving all magnitude information. Rotated experimental data for the complex current harmonics are designated ${Ĩ}_{j,\mathrm{rot}}$ .

The final data processing step is removal of the nonfaradaic capacitive currents that appear in all the admittance experimental data. Parallel faradaic and nonfaradaic pathways across the electrochemical interface generates additive currents in the admittance experiments, so the final rotated and capacitive-current-free faradaic harmonics, denoted ${Ĩ}_{j,\mathrm{proc}}$ , are given by

3 $${Ĩ}_{1,\mathrm{proc}}={Ĩ}_{1,\mathrm{rot}}+i\times C^{\mathrm{dl}}\times \omega \times \Delta E$$

and

4 $${\tilde{I}}_{j,\mathrm{proc}}={\tilde{I}}_{j,\mathrm{rot}},\mathrm{for}\;j\geq 2$$

where *C*_*dl*_ is the double layer capacitance (Farad) and *i* is the imaginary unit. Here we assume the small capacitive current has a linear response and therefore only affects the first harmonic signal at *j* = 1, which is only rigorously true for a Helmholtz-like double layer. An experimental best-fit capacitance *C*_*dl,exp*_ = 1.21 × 10^–5^ F is used to remove capacitive contributions from all the first harmonic currents analyzed here. See Figures S1 and S2 and ref 26 for the effects of these experimental data transformations.

## Results and Discussion

### Weakly Nonlinear Theory for the Warburg Admittance

In electroanalytical chemistry, a common reversible redox couple is dilute ferri-/ferro-cyanide in well-supported electrolyte, measured on a platinum working electrode

5 $$\mathrm{Fe}\left(\mathrm{III}\right)+e^{-}⇌\mathrm{Fe}\left(\mathrm{II}\right)$$

where, for shorthand, we only denote the redox couple’s iron oxidation state. As noted, for Warburg analysis, faradaic rates are governed by Fickian diffusion (a linear governing equation) coupled to Nernstian thermodynamics (a nonlinear algebraic equation). Fick’s second law is given by

6 $$\frac{\partial c}{\partial t}=D\frac{\partial^{2}c}{\partial x^{2}}$$

with Warburg analysis assuming sinusoidal concentration modulation at the electrode surface (*x* = 0) given by

7 $$c=c^{b}+\frac{C_{j}^{s}}{2}\times \left(e^{\mathrm{ij}\omega t}+e^{-\mathrm{ij}\omega t}\right),\mathrm{at}\;x=0$$

and a far-field bulk concentration of

8 $$c=c^{b},\mathrm{at}\;x\to \infty$$

Here *c*(*x*,*t*) denotes the concentration of the Fe­(II) or Fe­(III) species (mol/m^3^) in the electrolyte, *x* is the distance from the electrode’s surface (m), *t* is time (s), *D* is the diffusion coefficient of the Fe­(II) or Fe­(III) species (m^2^/s), and *c* ^*b*^ is the constant bulk concentration far from the electrode’s surface (mol/m^3^). Note that *i* defines the imaginary unit while *j* is the harmonic integer. As a linear equation, concentration fluctuations at any frequency are independent of all other frequencies. So, a subtle point in eq 7 is that the variable *C*_*j*_^*s*^ is the real magnitude of surface concentration oscillations (mol/m^3^) at the system excitation frequency ω (*j* = 1) and any harmonics (*j* = 2, 3, ...) that might be excited by the modulation of potential. Finally, to match our equimolar experimental data, *c* ^*b*^ is identical for both Fe species.

The analytical solution for eqs 6–8 involves the linear superposition of all oscillating surface concentration harmonics *j* ^27^

9 $$c\left(x,t\right)=c^{b}+\sum \left(\frac{C_{j}^{s}}{2}\times e^{-\left(1+i\right)\sqrt{\frac{j\omega }{2D}}\times x}\times e^{\mathrm{ij}\omega t}+\frac{C_{j}^{s}}{2}\times e^{-\left(1-i\right)\sqrt{\frac{j\omega }{2D}}\times x}\times e^{-\mathrm{ij}\omega t}\right)$$

where $\sqrt{\frac{2D}{j\omega }}$ defines the fluctuating diffusion layer thickness. The diffusion-limited faradaic current is given by the flux boundary condition at the electrode and Faraday’s law

10 $$I\left(t\right)=\mathrm{FAD}{\frac{\partial c\left(x,t\right)}{\partial x}|}_{x=0}$$

where *F* is the Faraday constant (96,485 C/mol), *A* is the area of the electrode interface (m^2^), and we have assumed a one-electron transfer per eq 5. Defining the oxidation current as positive and the reduction current as negative, the steady periodic current generated by each surface concentrations harmonic can be written using eqs 9 and 10 as

11 $$I_{j}\left(t\right)=-\mathrm{FA}{C_{j}}^{s,\mathrm{II}}\sqrt{\frac{{j\omega D}^{\mathrm{II}}}{2}}\times \frac{1}{2}\left[e^{\mathrm{ji}\omega t}\left(1+i\right)+e^{-\mathrm{ji}\omega t}\left(1-i\right)\right]$$

12 $$I_{j}\left(t\right)=\mathrm{FA}{C_{j}}^{s,\mathrm{III}}\sqrt{\frac{{j\omega D}^{\mathrm{III}}}{2}}\times \frac{1}{2}\left[e^{\mathrm{ji}\omega t}\left(1+i\right)+e^{-\mathrm{ji}\omega t}\left(1-i\right)\right]$$

where *D* ^*II*^ is the ferrocyanide species diffusion coefficient (m^2^/s) and *D* ^*III*^ is the ferricyanide species diffusion coefficient (m^2^/s). Equations 11 and 12 are equal, so the complex current harmonic coefficient in the frequency domain ( ${Ĩ}_{j}$ ) can be defined as

13 $${Ĩ}_{j}=\mathrm{FA}C_{j}^{s,\mathrm{III}}\sqrt{\frac{j\omega D^{\mathrm{III}}}{2}}\times \frac{1}{2}\left(1+i\right)=-\mathrm{FA}C_{j}^{s,\mathrm{II}}\sqrt{\frac{j\omega D^{\mathrm{II}}}{2}}\times \frac{1}{2}\left(1+i\right)$$

for the positive frequency term, with the corresponding complex conjugate multiplying the negative frequency term. Experimentally, it is common in signal processing to attribute the entire spectral power to the positive frequencies, with eq 13 multiplied by 2×. When working with Gamry experimental harmonic current data, this factor of 2× is required, so we included it in all final equations used for experimental analysis, below. The Supporting Information retains all positive and negative frequency terms and conjugates for mathematical correctness.^26^

Eliminating common factors in eq 13 reveals how the magnitude of the fluctuating surface concentrations for ferri- and ferro-cyanide are related to their individual diffusivities

14 $${C_{j}}^{s,\mathrm{II}}=-{C_{j}}^{s,\mathrm{III}}\times \sqrt{\frac{D^{\mathrm{III}}}{D^{\mathrm{II}}}}$$

The negative sign in eq 14 shows that the concentration fluctuations are 180° out of phase for reactant and product, as expected based on reaction eq 5.

So far, we have dealt with the linear equation governing diffusion-limited faradaic current, recognizing that a potential modulation will result in an oscillating surface concentration harmonic spectrum for each redox species. For an electrochemical interface governed by the Nernst equation, one can show that the relationship between surface concentrations, the electrode potential (*E*), and the stationary Nernst potential (*E*_*n*_) in our equimolar bulk electrolyte is

15 $$\frac{c^{s,\mathrm{III}}}{c^{s,\mathrm{II}}}=e^{f\left(E-E_{n}\right)}$$

where *c* ^*s*,*III*^ is the surface concentration of ferricyanide, *c* ^*s*,*II*^ is the surface concentration of ferrocyanide, and *f* = $\frac{F}{\mathrm{RT}}$ . Assuming a weakly nonlinear response with *f* × (*E* – *E*_*n*_) ≪ 1, eq 15 can be expanded as a series

16 $$\frac{c^{s,\mathrm{III}}}{c^{s,\mathrm{II}}}=1+f\left(E-E_{n}\right)+\frac{1}{2}f^{2}{\left(E-E_{n}\right)}^{2}+\mathrm{h.o.t.}$$

where *h.o.t.* represents the higher-order terms beyond those shown. Equations 15 and 16 are time-invariant thermodynamic expressions, so we can insert the cosine potential modulation used experimentally, namely,

17 $$E-E_{n}=\Delta E\times \cos\left(\omega t\right)=\frac{\Delta E}{2}\times \left(e^{i\omega t}+e^{-i\omega t}\right)$$

in eq 16 to approximate the fluctuating surface concentration ratio ( $\frac{c^{s,\mathrm{III}}}{c^{s,\mathrm{II}}}$ ) in the weakly nonlinear regime as

18 $$\frac{c^{s,\mathrm{III}}}{c^{s,\mathrm{II}}}=\left[1+\frac{f^{2}{\Delta E}^{2}}{4}\right]+\frac{1}{2}\;f\Delta E\times \left(e^{i\omega t}+e^{-i\omega t}\right)+\frac{1}{8}\;f^{2}{\Delta E}^{2}\times \left(e^{i2\omega t}+e^{-i2\omega t}\right)+\mathrm{h.o.t.}$$

where Δ*E* is the peak amplitude of the purely real input potential modulation (V). Equations 7, 14 and 18 define a set of real valued equations for each harmonic coefficient multiplying (*e* ^*ijωt*^ + *e* ^–*ijωt*^). Through substantial algebraic manipulation of these harmonic coefficient equations, and simplifications facilitated by the fact that *C*_*j*_^*s*^ ≪ *c* ^*b*^ for weakly nonlinear modulations that are consistent with eq 16, it is possible to define the modulation-potential-dependent surface concentration coefficients for ferri- and ferro-cyanide (*C*_*j*_^*s*,*III*^ and *C*_*j*_^*s*,*II*^). This algebraic manipulation is presented in a tutorial Jupyter Notebook^28^ in ref 26.

To further illustrate the role of diffusion coefficients in breaking the inversion symmetry that controls which harmonics are active, we explicitly assume a redox couple with similar diffusivities, so one can write

19 $$D^{\mathrm{II}}=D^{0}\left(1-\delta \right)$$

and

20 $$D^{\mathrm{III}}=D^{0}\left(1+\delta \right)$$

where *D* ^0^ is the mean diffusion coefficient (m^2^/s), and the diffusivity asymmetry parameter δ can be a small positive or negative number compared to unity (|δ| ≪ 1).

The modulation-potential-dependent *C*_*j*_^*s*,*III*^ coefficients (solved in ref 26) were inserted into eq 13 and simplified to find the following leading-order models for the complex first $({Ĩ}_{1}),$ second ( ${Ĩ}_{2}$ ), and third ( ${Ĩ}_{3}$ ) harmonic diffusion-limited faradaic current coefficients

21 $${Ĩ}_{1}=\frac{\mathrm{FA}c^{b}}{2}\sqrt{\frac{D^{0}\times \omega }{2}}\times \left(1+i\right)\times f\times \Delta E$$

22 $${Ĩ}_{2}=-\delta \times \frac{\mathrm{FA}c^{b}}{16}\times \sqrt{D^{0}\times \omega }\times \left(1+i\right)\times f^{2}\times {\Delta E}^{2}$$

and

23 $${Ĩ}_{3}=-\frac{\mathrm{FA}c^{b}}{192}\sqrt{D^{0}\times 6\omega }\times \left(1+i\right)\times f^{3}\times {\Delta E}^{3}$$

Note that each leading-order harmonic current ${Ĩ}_{j}$ grows as (*f* × Δ*E*)^*j*^, and when the two diffusivities are identical (δ = 0), the second harmonic (and, not shown, all other even harmonics) are identically zero. In short, equal diffusivities generate an electrochemical center of inversion symmetry around the stationary equilibrium potential and only odd harmonics exist in the resulting current spectrum. Differences in diffusivity generate a full spectrum with even and odd harmonics present.

### Higher-Order Nonlinear Correction Terms

In this section, we sequentially add weakly nonlinear correction terms to the leading-order first and second harmonic currents to increase their accuracy as *f* × Δ*E* approaches unity. The Python package *Sympy* was used to expand, filter, and rearrange terms into the desired forms for use with eq 13 (see ref 26). The fourth-order corrected models for the first and second harmonic currents are

24 $${Ĩ}_{1}=\frac{\mathrm{FA}c^{b}}{2}\sqrt{\frac{D^{0}\times \omega }{2}}\times \left(1+i\right)\times f\times \left[1-\frac{f^{2}{\Delta E}^{2}}{16}+\epsilon \left(f^{4}{\Delta E}^{4}\right)\right]\times \Delta E$$

and

25 $${Ĩ}_{2}=-\delta \times \frac{\mathrm{FA}c^{b}}{16}\sqrt{D^{0}\times \omega }\times \left(1+i\right)\times f^{2}\times \left[1-\frac{7\times f^{2}{\Delta E}^{2}}{24}+\epsilon \left(f^{4}{\Delta E}^{4}\right)\right]\times {\Delta E}^{2}$$

while the sixth-order corrected models are

26 $${Ĩ}_{1}=\frac{\mathrm{FA}c^{b}}{2}\sqrt{\frac{D^{0}\times \omega }{2}}\times \left(1+i\right)\times f\times \left[1-\frac{f^{2}{\Delta E}^{2}}{16}+\frac{f^{4}{\Delta E}^{4}}{192}+\epsilon \left(f^{6}{\Delta E}^{6}\right)\right]\times \Delta E$$

and

27 $${Ĩ}_{2}=-\delta \times \frac{\mathrm{FA}c^{b}}{16}\sqrt{D^{0}\times \omega }\times \left(1+i\right)\times f^{2}\times \left[1-\frac{7\times f^{2}{\Delta E}^{2}}{24}+\frac{19\times f^{4}{\Delta E}^{4}}{256}+\epsilon \left(f^{6}{\Delta E}^{6}\right)\right]\times {\Delta E}^{2}$$

In eqs 24–27, we explicitly show the scaling of error, ϵ, associated with truncating at specific model orders. Note that each ordered model rigorously only describes behavior in the weakly nonlinear regime, where *f* × Δ*E* ≪ 1. This is an inherent limitation of our theory that arises from a convergence criterion in an underlying Taylor series used to derive *C*_*j*_^*s*,*III*^ and *C*_*j*_^*s*,*II*^. See ref 26 for more information.

The only two unknowns in eqs 21–27 are the diffusion asymmetry parameter (δ) and mean diffusion coefficient (*D* ^0^), given that the bulk concentration (*c* ^*b*^), frequency (ω), and *f* = *F*/*RT* are generally known to the experimentalist. The lumped parameter that is preserved across these equations

28 $$K_{W}=\frac{\mathrm{FA}c^{b}\sqrt{D^{0}}}{2}$$

is labeled the Warburg coefficient, with units of (A·s^1/2^).

### Generalized Model Functional Form

As shown above, weakly nonlinear theory for the first harmonic faradaic current has a separable functional form

29 $${Ĩ}_{1}=K_{W}\times {\mathrm{g̃}}_{1}\left(\omega \right)\times h_{1}\left(\Delta E\right)\times \Delta E$$

where ${\mathrm{g̃}}_{1}(\omega )$ is a complex frequency-dependent function common among eqs 21, 24 and 26. The function *h*_1_(Δ*E*) is unity in the leading-order solution, eq 21, but has additional modulation-dependent terms as the correction order increases in eqs 24 and 26. The structure of the weakly nonlinear theory for the complex second harmonic faradaic current is also separable, with the form

30 $${Ĩ}_{2}=-\delta \times K_{W}\times {\mathrm{g̃}}_{2}\left(\omega \right)\times h_{2}\left(\Delta E\right)\times \Delta E^{2}$$

where ${\mathrm{g̃}}_{2}(\omega )$ is a complex frequency-dependent function common among eqs 22, 25 and 27. The function *h*_2_(Δ*E*) is unity for the leading-order solution, eq 22, but has additional modulation-dependent terms as the correction order increases in eqs 25 and 27. The dimensionless modulation amplitude dependent functions *h*_1_(Δ*E*) and *h*_2_(Δ*E*) have no fitting parameters, and are summarized in Table 1.

**Table 1:** Weakly Nonlinear Correction Functions *h*_*j*_(Δ*E*) for Finite Amplitude Potential Modulations^a^

Order of Model	*h*_1_(Δ*E*)	*h*_2_(Δ*E*)
2nd	1	1

4th	$1-\frac{{\Delta E}^{2}f^{2}}{16}$	$1-\frac{7\times {\Delta E}^{2}f^{2}}{24}$

6th	$1-\frac{{\Delta E}^{2}f^{2}}{16}+\frac{{\Delta E}^{4}f^{4}}{192}$	$1-\frac{7\times {\Delta E}^{2}f^{2}}{24}+\frac{19\times {\Delta E}^{4}f^{4}}{256}$

a Separable amplitude-dependent nonlinear correction function derived from eqs 21, 22 and 24–27.

### Qualitative Exploration of Experimental Data

Figure 1 shows the stable (cycle 3) cyclic voltammogram of dilute ferri-/ferro-cyanide in excess KCl supporting electrolyte with a scan rate (ν) of 20 mV/s. The peak separation of 60 mV suggests a reversible system. Perfect inversion symmetry is defined by each point on a CV oxidation branch being equidistant from the origin as its corresponding point on the reduction branch of the voltammogram. In Figure 1, this means that any two points, such as the oxidation and reduction peaks, would have equal but opposite heights and voltage separation from the origin. Likewise, any random pair of points that bisect the origin would have equal lengths *a* and *a*′. The ferri-/ferro-cyanide voltammogram is close to having inversion symmetry, with a peak height difference of just over 1%. It is unclear if the small anodic and cathodic peak difference is attributable entirely to differences in species diffusivities or whether other experimental factors arise over a 600 mV window—variations in capacitive background, free-convection, or dioxygen reduction—to obscure the intrinsic diffusion limited current. Experimental literature suggests a larger difference in the species diffusivities than implied by the 1% peak difference.

**Figure 1:**
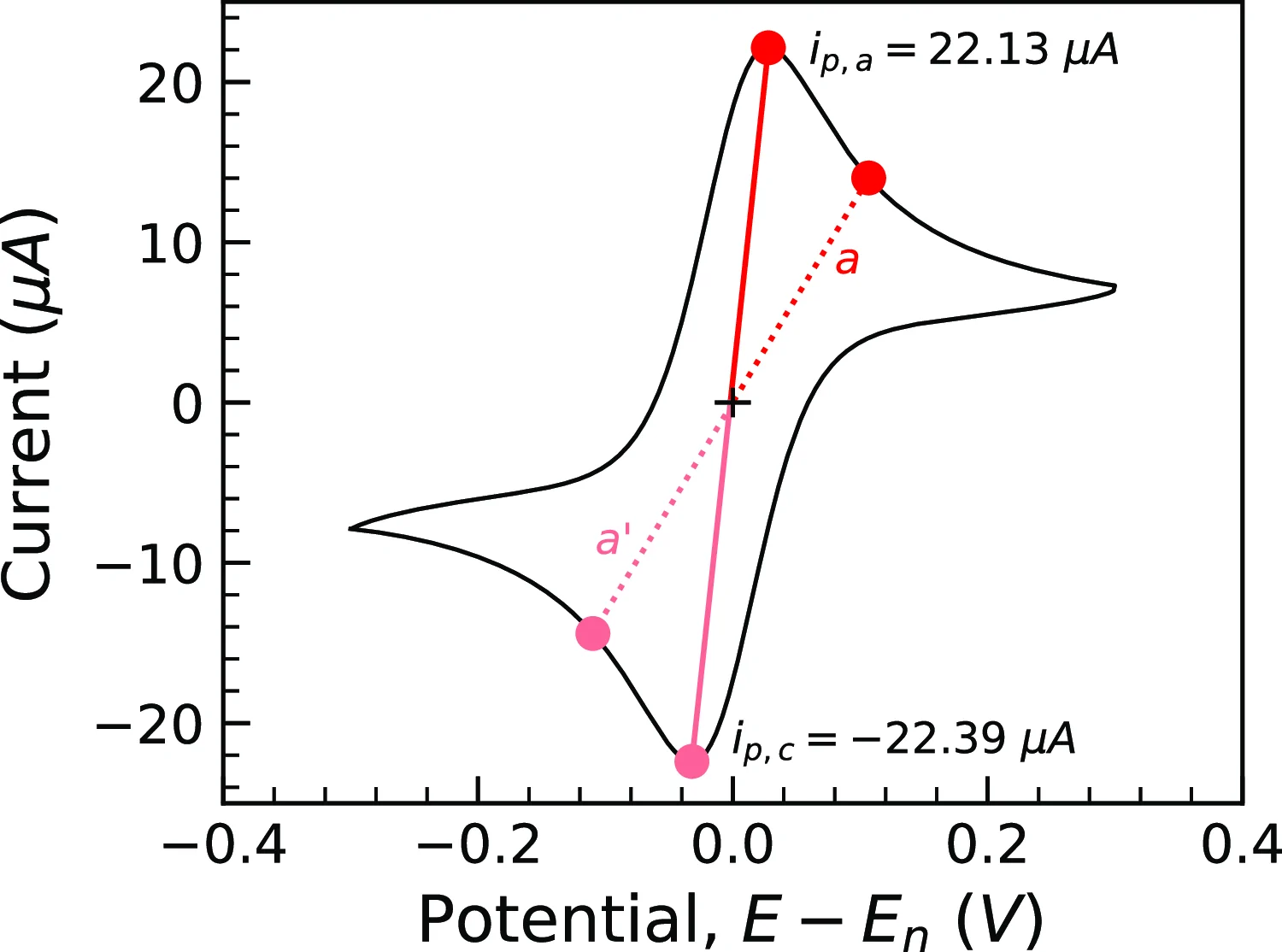
Cyclic voltammetry (Cycle 3) of equimolar ferri- and ferro-cyanide on Pt electrode. Electrolyte has a concentration of 1.923 mM for each redox species and 50 mM KCl support. Scan rate = 20 mV/s and *T* ≈ 25 °C. The anodic (*i*_*p*,*a*_) and cathodic (*i*_*p*,*c*_) peaks are located at (27.93 mV, 22.13 μA) and (−32.08 mV, –22.39 μA), respectively, and are connected by a line that passes near the origin (solid line). For illustrative purposes, a second arbitrary line is drawn through the origin (dotted line), intersecting each branch at (106.71 mV, 14.01 μA) and (−109.78 mV, –14.41 μA). These small differences are preliminary indications of asymmetry that may drive even harmonics.

As the theory above shows, electrochemical admittance measurements in the weakly nonlinear regime let us explore the individual species diffusivities using modest potential deviations from equilibrium. Figure 2 plots the processed harmonic current data delivered by the potentiostat for the equimolar ferri-/ferro-cyanide experiments with potential modulation at 0.3 Hz, corresponding to the midpoint of the 12 sampled frequencies, and peak amplitudes of 1.414 mV, 7.071 mV, and 14.142 mV. In Goh et al., all three of these modulation amplitudes would be deemed sufficiently linear for conventional Warburg analysis.^24^

**Figure 2:**
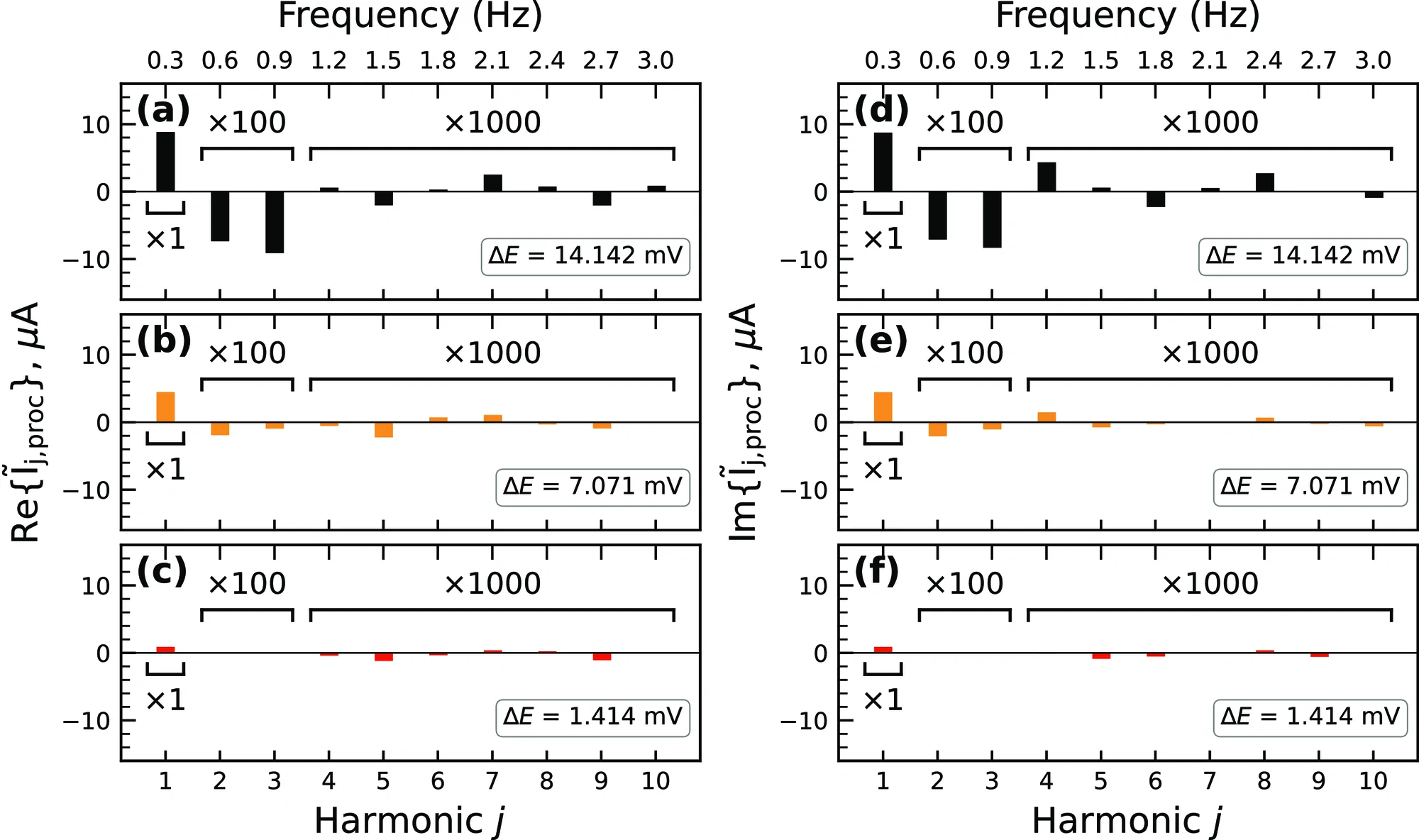
Experimental current harmonics ( ${Ĩ}_{j,\mathrm{proc}}$ ) for potential modulations at a fundamental frequency of 0.3 Hz. Subplots a–c and d–f depict the real and imaginary processed current output (μA), respectively, for the first ten harmonics (*j* = 1 to 10) generated by 14.142 mV (a,d), 7.071 mV (b,e) and 1.414 mV (c,f) peak single-sine potential modulations at 0.3 Hz. The small higher harmonic (*j* ≥ 2) signals are magnified by the × label above each bar to improve resolution against the *y*-axis.

The 0.3 Hz single-sine potential modulation generates measurable first, second, and third current harmonics, at 0.3, 0.6, and 0.9 Hz, respectively. The harmonics are seen to have comparable real and imaginary components for potential modulations ≥7.071 mV (note second and third harmonics are amplified 100× to make them visible on the same scale). This agrees with the (1 + *i*) notation for faradaic current in eqs 21–23, where each real and imaginary component is equal. Further, the positive sign in eq 21 and negative sign in eqs 22 and 23 agree with the bar directions of the measured first, second and third current harmonics. Note that had we neglected the experimental vector rotation associated with the phase of the experimental input potential, e.g., eqs 1 and 2, the observed relationship between real and imaginary output current would be lost. These transformation effects were also confirmed for the other 11 sampled frequencies. In all our work, harmonic currents with less than 1 nA magnitude were considered too noisy for quantitative analysis. More on this will be discussed later.

The magnitudes of signal growth for the first three harmonics (*j* = 1, 2, 3) from Figure 2 were examined on a log–log plot against the potential modulations probed experimentally (1.414 mV ≤ Δ*E* ≤ 141.421 mV) to show the full range of nonlinear effects. Figure 3 depicts the log-forms of eqs 21–23, where the respective slope of each leading-order model is proportional to its harmonic integer *j*. Leading-order models were fit to the two smallest measurable current amplitudes for each harmonic, as denoted by the filled symbols, and were extrapolated to high modulations where we expect leading-order solutions to increasingly deviate from experiments (denoted by open symbols in Figure 3).

**Figure 3:**
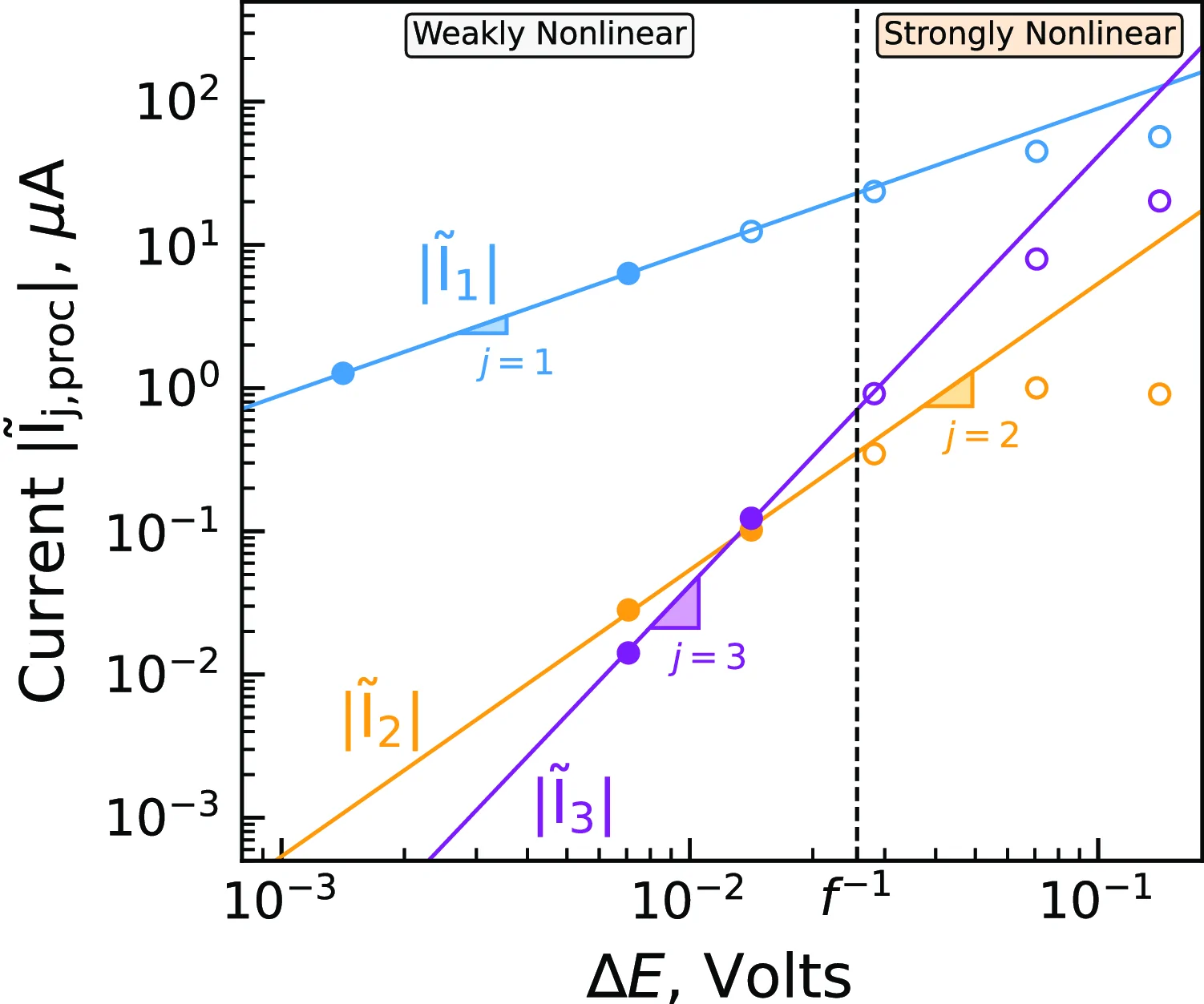
Experimental and leading-order models of current magnitudes $\left|{Ĩ}_{j}\right|$ versus potential modulation (Δ*E*) at 0.3 Hz. Experimental $\left|{Ĩ}_{j,\mathrm{proc}}\right|$ (circle markers) and leading-order model fits (solid lines) are shown for the first (blue), second (orange), and third (purple) harmonics, with the slope *j* indicated by a triangle. Filled markers denote the experiment used in fitting. The dotted line at *f* ^–1^ = $\frac{\mathrm{RT}}{F}$ marks the nominal boundary of the weakly nonlinear regime (*f* × Δ*E*) < 1.

In Figure 3, experimental points show that the first harmonic (*j* = 1) follows the linear leading-order model closely in the 1.414 mV ≤ Δ*E* ≤ 14.142 mV region, before systematically deviating below the leading-order theory at higher modulations. Similar deviations between experiments and leading-order theory when Δ*E* > 14.142 mV are seen for the quadratic second and cubic third harmonics leading-order models. These deviations arise because leading-order models neglect the additional contributions of higher-order nonlinear terms from the Nernst equation. The higher-order nonlinear correction terms in eqs 24–27 seek to address this issue for the first and second harmonic currents. Also shown in Figure 3 is the *f* ^–1^ line (near 26 mV) which denotes the modulation amplitudes below which weakly nonlinear analysis is expected to hold. Similar trends observed for the other 11 frequencies are omitted here for clarity but shown by Figures S6 and S7 in the Supporting Information.

### Model-Based Analysis of Experimental Data

To illustrate the nature of the weakly nonlinear admittance theory presented above, we explore the performance and information content of experimental data by separating the amplitude-dependent functions *h*_*j*_(Δ*E*) and complex frequency-dependent functions ${\mathrm{g̃}}_{j}(\omega )$ to show the “universal” behavior of the experimental data analyzed here.

We first explore how experimental data aligns with the nonlinear modulation functions *h*_*j*_(Δ*E*) in Table 1. Equation 29 shows that normalizing the magnitude of experimental first harmonic current with $\left|{\mathrm{g̃}}_{1}(\omega )\right|$ = $f\sqrt{\omega }$ should result in a “universal” amplitude-dependent curve that is free of all frequency dependencies. Figure 4a plots this frequency-normalized first harmonic current for the six potential modulation amplitudes studied. At each potential modulation amplitude, there are 24 superimposed measurements from 12 frequencies (<1.2 Hz) replicated twice. Each set of 24 experimental points superimpose beautifully on this log–log plot and look like a single point, especially within the weakly nonlinear regime (Δ*E* ≲ 28.284 mV). The points outside the weakly nonlinear regime (Δ*E* = 70.711 mV and 141.421 mV) also superimpose well, however. The outstanding scaling with $\left|{\mathrm{g̃}}_{1}(\omega )\right|$ , even at potential modulation amplitudes larger than 1/*f*, is largely explained by the fact that the frequency-dependent part of the faradaic current in eq 29 is governed by Fick’s second law, a linear equation. Plotted along with the normalized experimental points are the *h*_1_(Δ*E*) functions from Table 1, scaled to the data by setting *K*_*W*_ = 1.675 × 10^–5^ A·s^0.5^, the best fit of the sixth-order model function.

**Figure 4:**
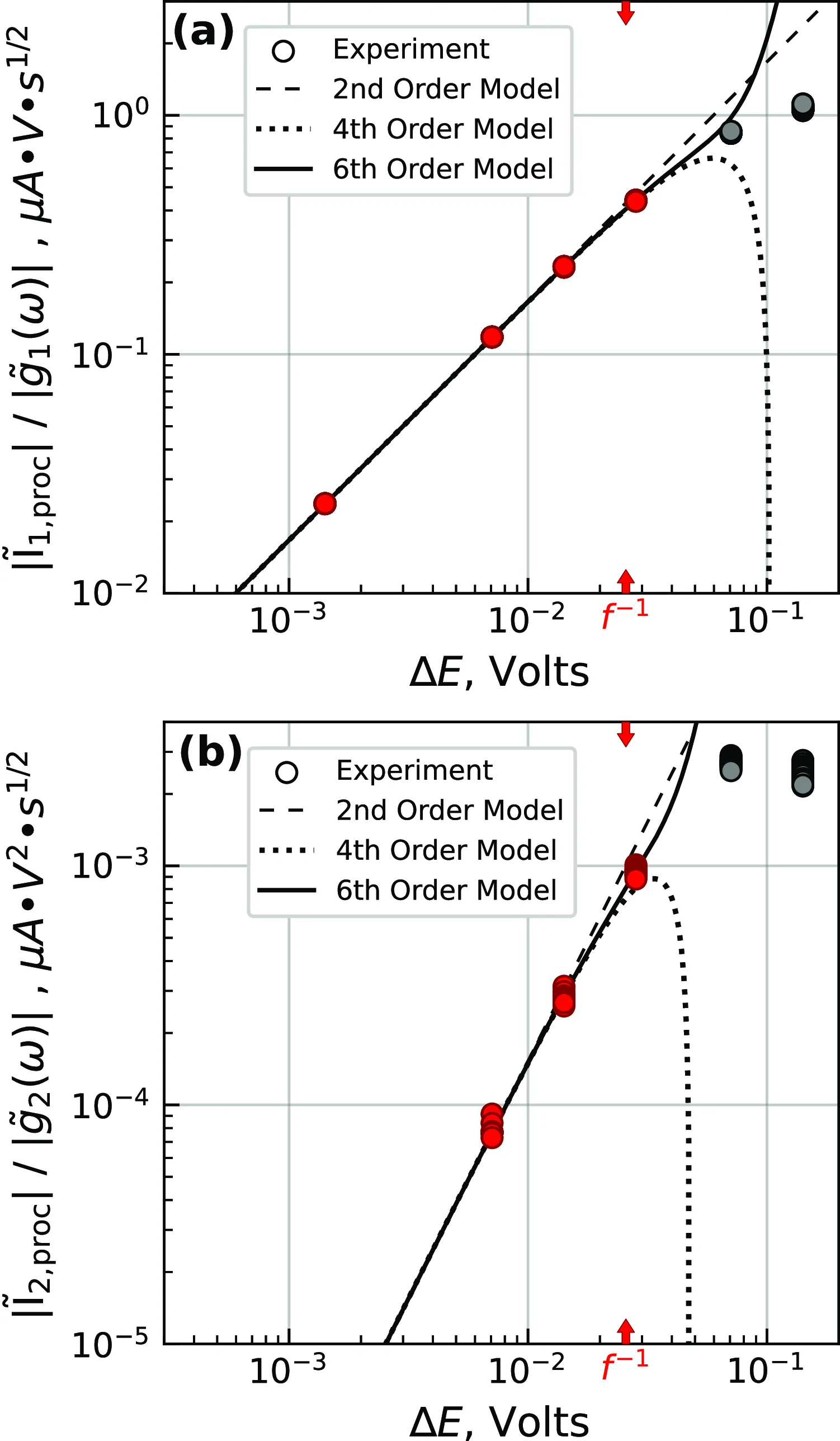
Frequency normalization of experimental first and second harmonic current reveals the “universal” modulation amplitude Dependence of each harmonic. Experimental frequency-normalized current magnitudes $\left|{Ĩ}_{j,\mathrm{proc}}\right|$ (circles) are compared to the leading-order model (dashed line) and corrected fourth-order (dotted line) and sixth-order (solid line) models for the (a) first harmonic and (b) second harmonic. Red filled circles denote the weakly nonlinear experiments, while gray filled circles denote the strongly nonlinear experiments well beyond *f* ^–1^ = $\frac{\mathrm{RT}}{F}$ (red arrows on top and bottom axes). Twenty-four points, from replicates of a dozen different experimental frequencies, are included at each modulation amplitude. Modeled curves all use *K*_*W*_ = 1.675 × 10^–5^ A·s^0.5^ and δ = 0.093.

Figure 4a shows that the leading-order, fourth-order, and sixth-order *h*_1_(Δ*E*) functions begin to diverge as modulation amplitude approaches and then exceeds 1/*f*. For Δ*E* < 28.284 mV, the models appear nearly indistinguishable on this log–log scale, overlapping with each other and the experiment. At Δ*E* = 28.284 mV, the two corrected models pass more accurately through the center of the experimental points, while the leading-order model overestimates the data. At higher amplitudes (Δ*E* = 70.711 mV and Δ*E* = 141.421 mV), the weakly nonlinear theory is not expected to hold, and we see the models deviating from the experimental observations. Yet, even at Δ*E* = 70.711 mV, the sixth-order correction seems to capture most behavior extremely well compared to the leading- and fourth-order functions.

For second harmonic current data, eq 30 suggests that one can remove all frequency dependence by plotting $\frac{\left|{Ĩ}_{2,\mathrm{proc}}\right|}{\left|{\mathrm{g̃}}_{2}\left(\omega \right)\right|}$ as a function of Δ*E*, where $\left|{\mathrm{g̃}}_{2}(\omega )\right|$ = $f^{2}\times \frac{\sqrt{2\omega }}{8}$ . Figure 4b shows the resulting frequency-normalized second harmonic current data plotted as a function of Δ*E*. It is noteworthy that the 24 experimental measurements at each value of Δ*E* do not collapse to a single indistinguishable overlapping point in Figure 4b, as they did in Figure 4a. In the next section, we will show why normalization of the second harmonic current by $\left|{\mathrm{g̃}}_{2}(\omega )\right|$ eliminates most, but not all, frequency dependence in the data. Plotted with the frequency-normalized second harmonic current data in Figure 4b are the leading-order, fourth-order, and sixth-order correction functions *h*_2_(Δ*E*) from Table 1, scaled by δ = 0.093. As with the first harmonic data, we see that all the functions largely overlap for Δ*E* < 28.284 mV, then increasingly diverge as the modulation amplitude approaches and exceeds 1/*f*, as denoted by the red arrows on the bottom and top axes. This divergence is intrinsic to the theory due to the use of truncated Taylor series in derivation. The direction each order of model diverges in the strongly nonlinear regime depends on sign of the last added term in each higher-order correction *h*_*j*_(Δ*E*): upward for the leading-order and sixth-order models (final correction term is positive-signed), and downward for the fourth-order model (final correction term is negative-signed).

Several features of the frequency-normalized harmonic currents presented in Figures 4 are of interest. Foremost, is that that the log–log plots emphasize the gross features of the curves as weakly nonlinear theory breaks down, but meaningful differences arising from nonlinearity exist and grow starting at 14.142 mV modulations. Next, in each plot, but especially Figure 4a, one sees frequency-normalized experimental data bends smoothly over the entire modulation range up to 141.421 mV, with only a little degradation in the quality of the frequency normalization. The implications of the observed smooth frequency-normalized data suggests that it may be possible to develop a holistic nonlinear theory for correcting the harmonic current data that enables extraction of the intrinsic first and second harmonic admittances at all modulation amplitudes. Whether using the approximate weakly nonlinear functions in Table 1, or a more holistic nonlinear theory, the method for analyzing admittance from the first and second harmonic data is identical to what we present below.

### Admittance-Based Parameter Estimation Using Leading-Order and Corrected Models

Rearranging eq 29 allows one to write a nonlinear-corrected admittance function that, in theory, removes all amplitude dependence from experimental first harmonic current data for any weakly nonlinear modulation

31 $${Ã}_{1}\left(\omega \right)=\frac{{Ĩ}_{1}\left(\omega ,\Delta E\right)}{h_{1}\left(\Delta E\right)\times \Delta E}=K_{W}\times {\mathrm{g̃}}_{1}\left(\omega \right)$$

where ${Ã}_{1}$ is the amplitude-independent first harmonic admittance (Ω^–1^), ${\mathrm{g̃}}_{1}(\omega )$ = $f\sqrt{\omega /2}\left(1+i\right)$ , and *h*_1_(Δ*E*) is an appropriate amplitude-dependent nonlinear correction function. To evaluate the admittance experimentally, we will use ${Ĩ}_{1,\mathrm{proc}}$ in eq 31 and the various analytical weakly nonlinear forms for *h*_1_(Δ*E*) in Table 1.

Rearranging eq 30 allows one to write a nonlinear-corrected second harmonic admittance function that, in theory, removes all the amplitude dependence from the experimental second harmonic current data for any weakly nonlinear modulation

32 $${Ã}_{2}\left(\omega \right)=\frac{{Ĩ}_{2}\left(\omega ,\Delta E\right)}{h_{2}\left(\Delta E\right)\times \Delta E^{2}}=-\delta \times K_{W}\times {\mathrm{g̃}}_{2}\left(\omega \right)$$

where ${Ã}_{2}$ is the second harmonic admittance (*A*/*V* ^2^), ${\mathrm{g̃}}_{2}(\omega )$ = $f^{2}\times \frac{\sqrt{\omega }}{8}\left(1+i\right)$ , and *h*_2_(Δ*E*) is an appropriate amplitude-dependent nonlinear correction function. To evaluate the admittance experimentally, we will use ${Ĩ}_{2,\mathrm{proc}}$ in eq 32 and the various analytical weakly nonlinear forms for *h*_2_(Δ*E*) in Table 1. The second harmonic admittance has a nearly identical form to the first harmonic admittance, though it is proportional to δ, so when the diffusivities are identical (δ = 0), the second harmonic admittance is zero owing to the electrochemical inversion symmetry of the system around the stationary equilibrium point.

The parameter *K*_*W*_ is found by fitting the admittance model *K*_*W*_ × ${\mathrm{g̃}}_{1}(\omega )$ from eq 31 to the experimental first harmonic admittance, ${Ã}_{1,\mathrm{proc}}$ = $\frac{{Ĩ}_{1,\mathrm{proc}}}{h_{1}\left(\Delta E\right)\times \Delta E}$ , estimated from single-sine measurements at discrete frequencies ω_*k*_. The squared error between experiments and model is minimized using *K*_*W*_ as the single fitting degree of freedom per

33 min⁡∑k=1K(Re{Ã1,proc}k−KW×Re{g̃1(ωk)})2+(Im{Ã1,proc}k−KW×Im{g̃1(ωk)})2

where the quality of our estimate for the experimental first harmonic admittance ${Ã}_{1,\mathrm{proc}}$ will depend on the function *h*_1_(Δ*E*) and the magnitude of the modulation. With *K*_*W*_ determined from the first harmonic response, the parameter δ is then found by fitting the second harmonic admittance model –δ × *K*_*W*_ × ${\mathrm{g̃}}_{2}(\omega )$ from eq 32 to the estimated experimental second harmonic admittance, ${Ã}_{2,\mathrm{proc}}$ = $\frac{{Ĩ}_{2,\mathrm{proc}}}{h_{2}\left(\Delta E\right)\times {\Delta E}^{2}}$ , measured at each discrete frequency ω_*k*_. In particular, the squared error is minimized using δ as the single fitting degree of freedom per

34 min⁡∑k=1K(Re{Ã2,proc}k+δ×Re{KW×g̃2(ωk)})2+(Im{Ã2,proc}k+δ×Im{KW×g̃2(ωk)})2

where the estimated experimental second harmonic admittance ${Ã}_{2,\mathrm{proc}}$ depends on our selection of the function *h*_2_(Δ*E*). For any given modulation amplitude, the number of experimental points we use to find the parameters for the first and second harmonic Warburg admittance is *K* = 24 (a pair of replicate experiments at 12 discrete frequencies).

Figure 5a is the experimental Nyquist plot for leading-order estimate (*h*_1_(Δ*E*) = 1) of the first harmonic Warburg admittance over the potential modulation range of 1.414 mV ≤ Δ*E* ≲ 28.284 mV where weakly nonlinear behavior is expected. Two types of uncertainty are reported. Error bars represent the 95% confidence interval of the minimized fits, computed as 1.96σ from the parameter covariance matrix. These intervals describe the sensitivity of each parameter to the data for a given model. The shaded gray bands represent ±1 standard deviation of the fitted parameters across modulation amplitudes, reflecting how well each model removes amplitude dependence. This uncertainty was used in error propagation where we expect amplitude dependence for estimated diffusion coefficients to be removed with correction. Only low-frequency points (<1.2 Hz) are presented to minimize capacitive and kinetic effects that may impact the results (the overlapping replicate experiments are not shown, for clarity). All experimental points fall on the 45° line, in accordance with the ${\mathrm{g̃}}_{1}(\omega )$ function in eq 31. Careful observation shows that the experimental points for 1.414 and 7.071 mV modulations overlap at all 12 frequencies in Figure 5a. However, the 14.142 mV modulation experimental points show a slight offset at every frequency compared to the 1.414 and 7.071 mV modulations, while the 28.284 mV points are substantially offset at all frequencies. The fit parameter *K*_*W*_ shows a modulation amplitude dependence in Figure 5b, where the 1.414 and 7.071 mV experimental values overlap (to within the fitting uncertainty), but at higher modulation amplitudes the values systematically decline; by 28.284 mV the fit value of *K*_*W*_ is more than 7% below the low-amplitude limiting values at 1.414 and 7.071 mV. Because the leading-order experimental estimate for the first harmonic admittance has no nonlinear corrections, it is only expected to rigorously hold in the infinitesimal modulation limit. Together, Figures 5a,b suggest that the rigorously linear limit is well represented by the 1.414 and 7.071 mV modulations, but then unaccounted nonlinearity leads to systematic and growing error in the estimated parameter as modulation amplitude increases.

**Figure 5:**
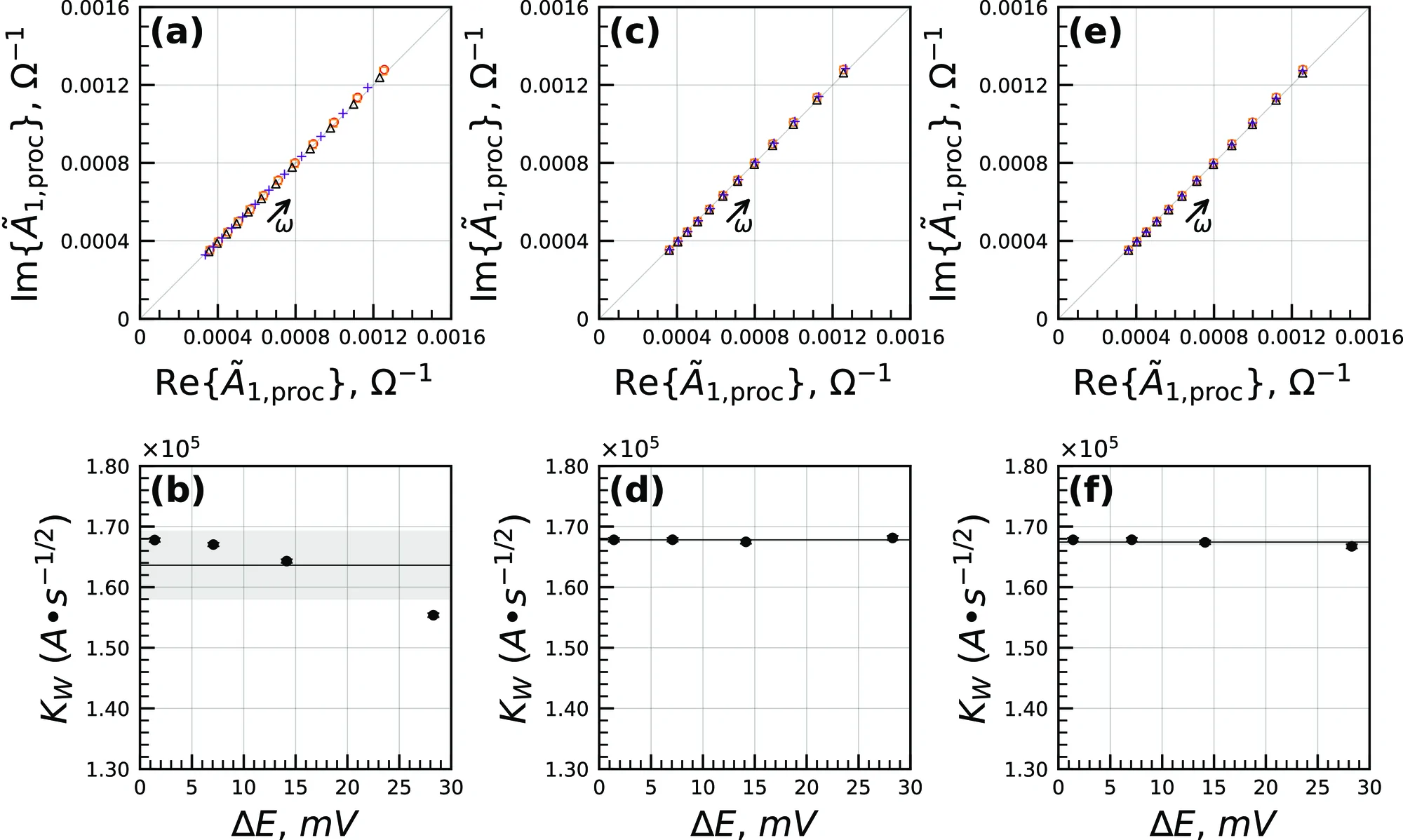
Impact of nonlinear corrections on the experimental first harmonic warburg admittance ${Ã}_{1}$ and fitted parameter *K*_*W*_. (a) Experimental first harmonic Warburg admittance values are estimated using the leading-order (linear) model, resulting in the (b) fit values of *K*_*W*_ for each modulation amplitude. Applying the (c) fourth-order or (e) sixth-order nonlinear corrections to the experimental data results in more tightly clustered experimental points on the Nyquist plot, and a reduction in amplitude dependence for the fits Warburg coefficients in (d) and (f). Experiment amplitudes are denoted by red circles (1.414 mV), orange squares (7.071 mV), black triangles (14.142 mV), and purple crosses (28.284 mV). Frequency increases along the direction of the overlaid arrows (<1.2 Hz). Replicates are excluded for clarity but included in model fitting. Fitting error bars from nonlinear least-squares for *K*_*W*_ (95% confidence interval, 1.96σ) are barely visible under symbols; solid black lines and gray shaded regions denote the mean and ±1 standard deviation of *K*_*W*_ for each model order.

Figures 5c,e show, respectively, the effect of correcting the experimental first harmonic Warburg admittance with the fourth- and sixth-order weakly nonlinear amplitude functions given in Table 1. In both Figures 5c and 5e the experimental data points cluster much more tightly at each frequency, for all amplitudes, compared to the data in Figure 5a. Moreover, the best fit value of *K*_*W*_ in Figures 5d,f shows a near-perfect correction of the modulation-amplitude dependence seen in Figure 5b, with a now strongly flattened curve. For any of the nonlinearly corrected models, *K*_*W*_ values estimated at each of the modulation amplitudes are essentially identical, with only minor variations that are within uncertainty.

If one is only interested in an accurate estimate of the system parameter *K*_*W*_, experiments with a modulation amplitude of 7.071 mV are ideal for producing a large experimental signal with an unmeasurable nonlinear contribution. However, the Warburg coefficient, *K*_*W*_, only allows one to determine the mean diffusivity of the redox pair, not the two individual species’ diffusivities. To independently determine both diffusivities from these admittance experiments requires analyzing the second harmonic data.

Figure 6a shows, for the first time, the leading-order (*h*_2_(Δ*E*) = 1) experimental second harmonic Warburg admittance for three values of potential modulation, 7.071, 14.142, and 28.284 mV (1.414 mV modulations did not produce measurable harmonic currents) and a dozen low-frequency points, with uncertainty reported in the same manner as Figure 5. In this case, all experimental data points are near the 45° theory line defined by the ${\mathrm{g̃}}_{2}(\omega )$ function in eq 32, but not directly on it. One observes amplitude-dependent offsets from the 45° line, where 7.071 mV data is above the line, 28.284 mV is below, and 14.142 mV is nearly on the 45° line. Obscured by the 45° offset is a more subtle amplitude-dependent spreading of points at each frequency, like what was observed in Figure 5a. Figure 6a shows that the leading-order model is under-corrective, with the largest modulation amplitude (28.284 mV) exhibiting a strong leftward offset, or curve compression, while smaller modulations (<28.284 mV) remain tightly clustered to the right at every frequency. In contrast, the fourth-order correction (Figure 6c) over-compensates, producing a strong rightward offset, or curve stretching, of the 28.284 mV modulation and an even tighter leftward clustering of the <28.284 mV modulations. The sixth-order model (Figure 6e) yields the most balanced correction, with modulation amplitudes collapsing more uniformly at each frequency. The remaining 45° vertical offset cannot be corrected by any of the ordered models and yet is responsible for the poorer “universal” behavior observed in Figure 4b compared to Figure 4a. This unexplained amplitude-dependent offset seems most likely to be attributable to signal processing, given that the data originating from the smallest signals (lowest frequency, smallest modulation amplitude points) display an aphysical “kink” in Figures 6a,c,e. Careful analysis of harmonic signal processing in the instrument software is warranted.

**Figure 6:**
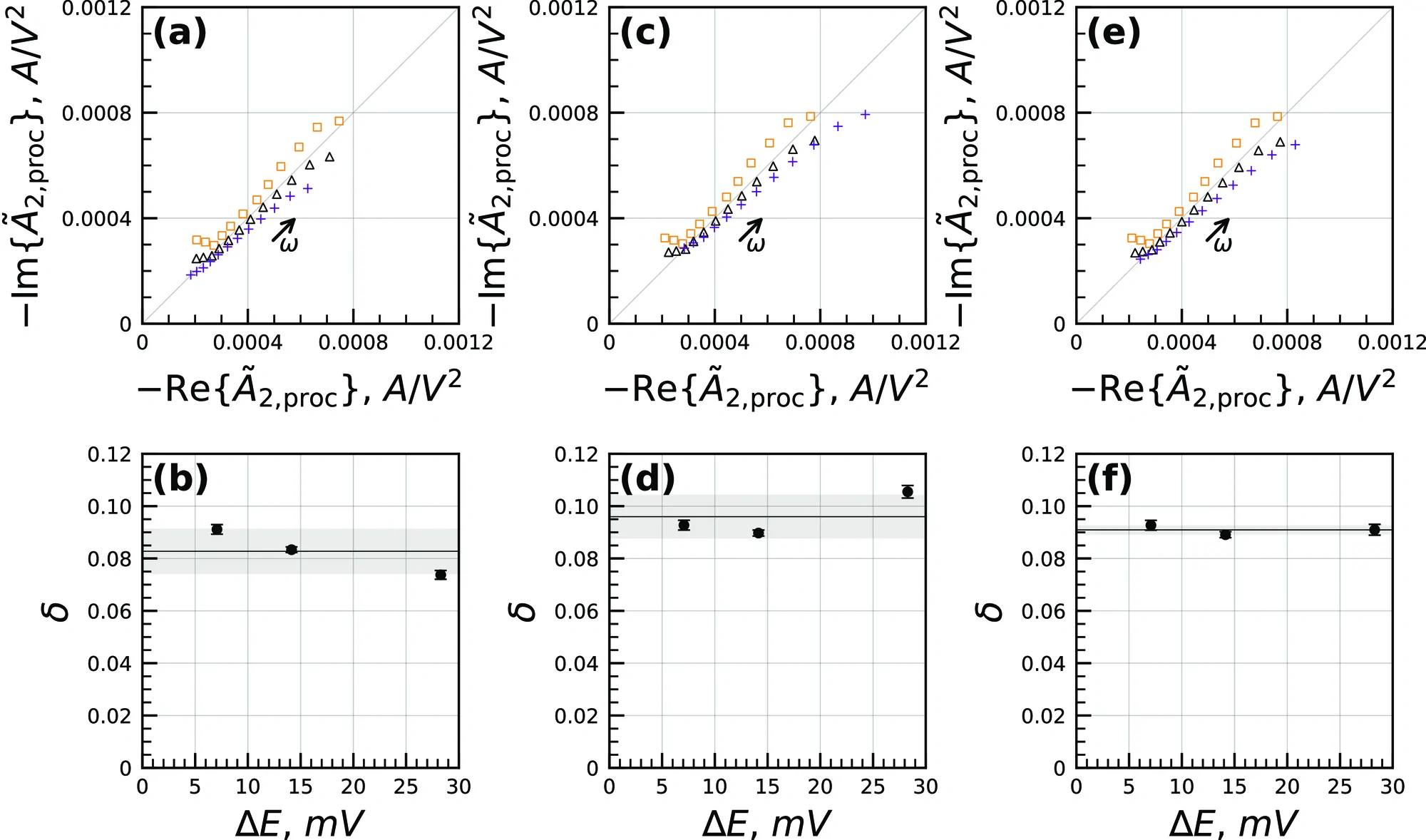
Impact of nonlinear corrections on the experimental second harmonic Warburg admittance ${Ã}_{2}$ and fitted parameter δ. (a) Experimental second harmonic Warburg admittance values are estimated using the leading-order (quadratic) model, resulting in the (b) fit values of diffusion asymmetry parameter δ for each modulation amplitude. Applying (c) fourth-order or (e) sixth-order nonlinear corrections to the second harmonic experimental data results in more tightly clustered experimental points on the Nyquist plot and a reduction in amplitude dependence for the fits of diffusion asymmetry parameters in (d) and (f). Experiment amplitudes are denoted by orange squares (7.071 mV), black triangles (14.142 mV), and purple crosses (28.284 mV). Frequency increases along the direction of the overlaid arrows (<1.2 Hz). Replicates are excluded for clarity but included in model fitting. Fitting error bars from nonlinear least-squares for δ (95% confidence interval, 1.96σ) are barely visible under symbols; solid black lines and gray shaded regions denote the mean and ±1 standard deviation of δ for each model order.

For each set of 24 experimental points in Figures 6a,c,e, we use the best-fit values of *K*_*W*_ from the corresponding first harmonic measurements to estimate the diffusivity asymmetry parameter δ, per eq 34. Thus, the leading-order experimental data in Figure 6a uses the corresponding values of *K*_*W*_ from Figure 5b to determine the best-fit values of δ shown in Figure 6b. Likewise, the nonlinearly corrected experimental data in Figures 6c,e utilize the *K*_*W*_ values in Figures 5d,f, respectively, to determine the best-fit values of δ in Figures 6d,f. The resulting best-fit values for δ in Figures 6b,d,f are fairly consistent at δ ≈ 0.09. In accordance with the observed left- and right-side offsets in Figures 6a,c and tighter modulation clustering in Figure 6e, Figure 6f shows that the sixth-order amplitude function removes almost all amplitude dependence from δ, with the estimation at 28.284 mV converging within 2% of the value at 7.071 mV, compared to only 14% and 19% for the leading- and fourth-order functions. While the δ estimates largely converge using corrected models, it is worth noting that there remain some systematic experimental offsets from the theoretical 45° line. This offset is likely due to a combination of signal processing issues for the small harmonic signals and the assumption that underpins eq 4, namely, an interfacial capacitive behavior akin to a Helmholtz double layer with no contribution to higher harmonics.

The inherently nonlinear electrochemical interface creates a trade-off between input modulation amplitude, output signal quality, and analysis complexity. Simple leading-order models are suitable for the smallest modulations (≤7.071 mV) where the second harmonic is measurable but with poor signal-to-baseline. Doubling the modulation amplitude quadruples the second harmonic signal, but nonlinearity begins to be evident in the fit parameters (Figures 5b and 6b), effects that are corrected with higher order (more complex) models; at 14.142 mV both the fourth- and sixth-order models adequately correct the experiments estimates for *K*_*W*_ and δ parameters (Figures 5d,f and 6d,f). Doubling modulation further to 28.28.4 mV continues this trend, where the sixth-order model is preferred for experimental fitting.

### Discussion of Fit Parameters

Based on the results above, values for the fit parameters *K*_*W*_ and δ acquired at a modulation of 14.142 mV represent a compromise between signal strength and quality of nonlinear correction of the data. Using the fits for the 14.142 mV data in Figures 5b,d,f and Figures 6b,d,f, one can compute the average diffusivity (*D* ^0^) and individual species diffusion coefficients (*D* ^*II*^, *D* ^*III*^) using eqs 19, 20, and 28. These results are summarized in Table 2.

**Table 2:** Diffusion Coefficients at Δ*E* = 10 mV_*rms*_ for Each Order of Model

Order of Model	*D* ^0^ (×10^6^ cm^2^/s)	*D* ^*II*^ (×10^6^ cm^2^/s)	*D* ^*III*^ (×10^6^ cm^2^/s)
2nd	6.3 ± 0.4	5.8 ± 0.4	6.8 ± 0.5
4th	6.52 ± 0.02	5.94 ± 0.06	7.11 ± 0.06
6th	6.52 ± 0.04	5.94 ± 0.04	7.10 ± 0.04

Errors were propagated using the standard deviations of *K*_*W*_ and δ across the fit modulation amplitudes (Figures 5 and 6), while constants (*F*, *R*, *T*, *c* ^*b*^, *A*) were assumed exact. The error generally decreases with correction order as the amplitude dependence is removed, agreeing with the narrowing gray bands in Figures 5 and 6.

In the literature, several approaches have been used to identify the ferri- and ferrocyanide diffusion coefficients, including, but not limited to, twin-electrode thin-layer electrochemistry, chronoamperometry, and microelectrode arrays.^7–11^ Depending on the concentration of KCl and ferri- and ferrocyanide species in the electrolyte, diffusion coefficients for *D* ^*II*^ and *D* ^*III*^ can vary widely. For a 1 M KCl electrolyte, the *D* ^*II*^ and *D* ^*III*^ coefficients ranged from [5.88–6.71] cm^2^/s and [6.77–7.74] cm^2^/s for varying concentrations of Fe species.^7–10^ From Table 2, the sixth-order estimates for each fall this range, respectively. Note that 50 mM KCl was used as supporting electrolyte here, a lower value than that from cited literature.

The cyclic voltammetry results in Figure 1 showed an ∼1% difference in peak heights, which results in an order of magnitude smaller difference in diffusivities compared to the δ ≈ 0.09 value found by this method. Our nonlinear admittance results are much more consistent with the literature than those found with CV. Perhaps because weakly nonlinear admittance experiments probe the system near the stationary equilibrium point (compared to a 600 mV CV sweep), complicating effects from gradient-induced free convection in the electrolyte and potential dependent capacitive changes are less likely to interfere in the measurement.

## Conclusions

This paper presented a framework for interpreting weakly nonlinear electrochemical Warburg admittance (Δ*E* ≲ *f* ^–1^) as a two-parameter extension of conventional one-parameter PEIS, enabling the unique determination of each species diffusivities in a redox couple. We emphasize the role of inversion-symmetry breaking in the second harmonic by parametrizing the problem based on redox species diffusion asymmetry. It was shown that the second harmonic has sufficient signal-to-noise for characterization at small modulation amplitudes (Δ*E* ≥ 7.071 mV), with the ability to extend the analysis to modulations Δ*E* ∼ *O*(*f* ^–1^) using correction terms. The sensitivity of nonlinear Warburg analysis to diffusion asymmetry makes this theory especially valuable for probing molecularly similar redox pairs where there are subtle conformational, charge, and solvation effects between the reduced and oxidized species, as is common in biological and synthetic macromolecular redox systems.

Data processing steps were shown for converting raw harmonic data acquired using the THD setting on a Gamry potentiostat to obtain the first 10 real and imaginary harmonics. Complex harmonic signals were converted to peak amplitudes and rotated to align the experimental phase behavior to theory, while a capacitive correction was used to remove capacitive currents from first harmonic response. Nonlinear capacitive effects were neglected.

More broadly, the second harmonic admittance is sensitive to all sources of electrochemical inversion asymmetry and can generate a full-spectrum response that is complementary to the linear response. Here we have focused on the asymmetry driven by differences in redox species diffusivities, but in future work we will introduce the frequency and amplitude dependences of asymmetry in interfacial charge transfer and capacitance and how they contribute to the second harmonic in both admittance and impedance experiments.

## Supplementary Material



## Data Availability

All code used in theory and model derivation (tutorial on solving for modulation-potential-dependent *C*_*j*_^*s*,*III*^ coefficients and correction functions (*h*_*j*_(Δ*E*)), parameter fitting (*K*_*W*_ and δ), and data used for all figure plotting is openly available via a Zenodo archive.^26^

## Notes

Comparison of rotated and processed first harmonic currents (Figure S1); experimental potential harmonics ( ${Ṽ}_{j,\mathrm{proc}}$ ) for 0.3 Hz (Figure S2); Nyquist plot of leading-order first harmonic admittances ${Ã}_{1,\mathrm{rot}}$ from 0.095 Hz to 1.5 MHz (Figure S3); zoomed-in Nyquist plots of leading-order first harmonic admittances ${Ã}_{1,\mathrm{rot}}$ near origin (Figure S4); zoomed-in Nyquist plots of leading-order second harmonic admittances ${Ã}_{2,\mathrm{rot}}$ near origin (Figure S5); replicate data for experimental leading-order models of current magnitudes $\left|{Ĩ}_{j}\right|$ versus potential modulation (Δ*E*) for the 12 smallest frequencies (Figures S6 and S7); replicate data for nonlinear corrections on the experimental first harmonic Warburg admittance ${Ã}_{1}$ and fitted parameter *K*_*W*_ (Figure S8); and replicate data for impact of nonlinear corrections on the experimental second harmonic Warburg admittance
  $${Ã}_{2}$$
  and fitted parameter δ (Figure S9) (PDF)

## Nomenclature

*F*: Faraday constant (96,485 C/mol·e^–^)
*R*: gas constant (8.314 J/mol·K)
*T*: temperature (K)
*f*: *F*/*RT* (V^–1^)
*j*: harmonic integer (unitless)
*c* ^*II*/*III*^(*x*,*t*): concentration of ferro-/ferri-cyanide (mol/m^3^)
*c* ^*b*^: equimolar bulk concentration of ferro-/ferri-cyanide (mol/m^3^)
*c*_*s*_^*II*/*III*^(*t*) = *c* ^*II*/*III*^(0,*t*): fluctuating surface concentration of ferro-/ferri-cyanide (mol/m^3^)
ω: fundamental frequency of applied potential signal (rad/s)
θ: phase angle of the first harmonic input potential modulation (rad)
*C*_*dl*_: double-layer capacitance (F)
*C*_*j*_^*II*/*III*^: real Fourier coefficient (mol/m^3^) for ferro-/ferri-cyanide species oscillating at integer harmonic frequency ω_*j*_ = *j* × ω
*D* ^0^ = $\frac{D^{\mathrm{II}}+D^{\mathrm{III}}}{2}$: mean bulk diffusion coefficient (m^2^/s)
*D* ^*II*^ = *D* ^0^(1 – δ): ferrocyanide species diffusion coefficient (m^2^/s)
*D* ^*III*^ = *D* ^0^(1 + δ): ferricyanide species diffusion coefficient (m^2^/s)
δ: diffusion asymmetry (|δ| ≪ 1) between ferri- and ferro-cyanide (unitless)
*A*: surface area of disk electrode (m^2^)
*K_W_* = $\frac{\mathrm{FA}c^{b}\sqrt{D^{0}}}{2}$: Warburg coefficient (A·s^1/2^)
Δ*E*_*rms*_: root-mean-square amplitude of the purely real input potential modulation for single-cosine experiments, the notation typically reported by instrument (V)
Δ*E* = √2 × Δ*E*_*rms*_: peak amplitude of the purely real input potential modulation for single-cosine experiments, the notation used by the theory (V)
${\mathrm{g̃}}_{1}(\omega )$ = $f\sqrt{\frac{\omega }{2}}\left(1+i\right)$: complex frequency-dependent function for first harmonic models (ω^1/2^·V^–1^)
${\mathrm{g̃}}_{2}(\omega )$ = $f^{2}\times \frac{\sqrt{\omega }}{8}\left(1+i\right)$: complex frequency-dependent function for second harmonic models (ω^1/2^·V^–2^)
*h*_1_(Δ*E*): modulation-amplitude-dependent functions for higher-order corrected first harmonic models (Table 1) (unitless)
*h*_2_(Δ*E*): modulation-amplitude-dependent functions for higher-order corrected second harmonic models (Table 1) (unitless)
${Ĩ}_{j}(\omega ,\Delta E)$: complex frequency- and potential-dependent diffusion-limited faradaic current coefficient oscillating at harmonic *j* (A)
${Ã}_{j}(\omega )$: complex frequency-dependent admittance oscillating at harmonic *j* (A·V^–*j*^)
