# Supplementary material for: Second Harmonic Nonlinear Warburg Admittance Analysis Eliminates Information Loss from Linearization in Traditional EIS: Theory and Experimental Validation
Source: Anal Chem. 2026 Aug 5;98(33):24057–69. doi: 10.1021/acs.analchem.6c01245 (PMC13523557; doi:10.1021/acs.analchem.6c01245)
Supplement: Supplementary file 1 [file ac6c01245_si_001.pdf]

Supporting Information

Second Harmonic Nonlinear Warburg Admittance Analysis Eliminates Information Loss from Linearization in Traditional EIS: Theory and Experimental Validation

Lauren A. Frank,[1] Jerome T. Babauta,[2] and Daniel T. Schwartz[1,*]

[1]Department of Chemical Engineering and
Clean Energy Institute
Box 351750
University of Washington
Seattle, WA 98195-1750

[2]Gamry Instruments, Inc.
734 Louis Drive
Warminster, PA 18974

Email: dts@uw.edu

Table of Contents

Figure S1 - Comparison of Rotated and Processed First Harmonic Currents S-2

Figure S2 - Experimental Potential Harmonics at Fundamental Frequency of 0.3 Hz S-3

Figure S3 - Nyquist Plot of Leading-Order First Harmonic Admittances (0.095 Hz–1.5 MHz)....................S-3

Figure S4 - Zoomed-In Nyquist Plots of Leading-order First Harmonic Admittances Near Origin S-4

Figure S5 - Zoomed-In Nyquist Plots of Leading-order Second Harmonic Admittances Near Origin....S-5

Figure S6 - Run 1 Replicate Data for Experimental Leading-Order Models of Current Magnitudes Versus Potential Modulation at 12 Smallest Frequencies S-6

Figure S7 - Run 2 Replicate Data for Experimental Leading-Order Models of Current Magnitudes Versus Potential Modulation at 12 Smallest Frequencies S-7

Figure S8 - Replicate Data for Nonlinear Corrections on the Experimental First Harmonic Warburg Admittance and Fitted Parameter K_W S-8

Figure S9 - Replicate Data for Impact of Nonlinear Corrections on the Experimental Second Harmonic Warburg Admittance and Fitted Parameter δ....................S-9

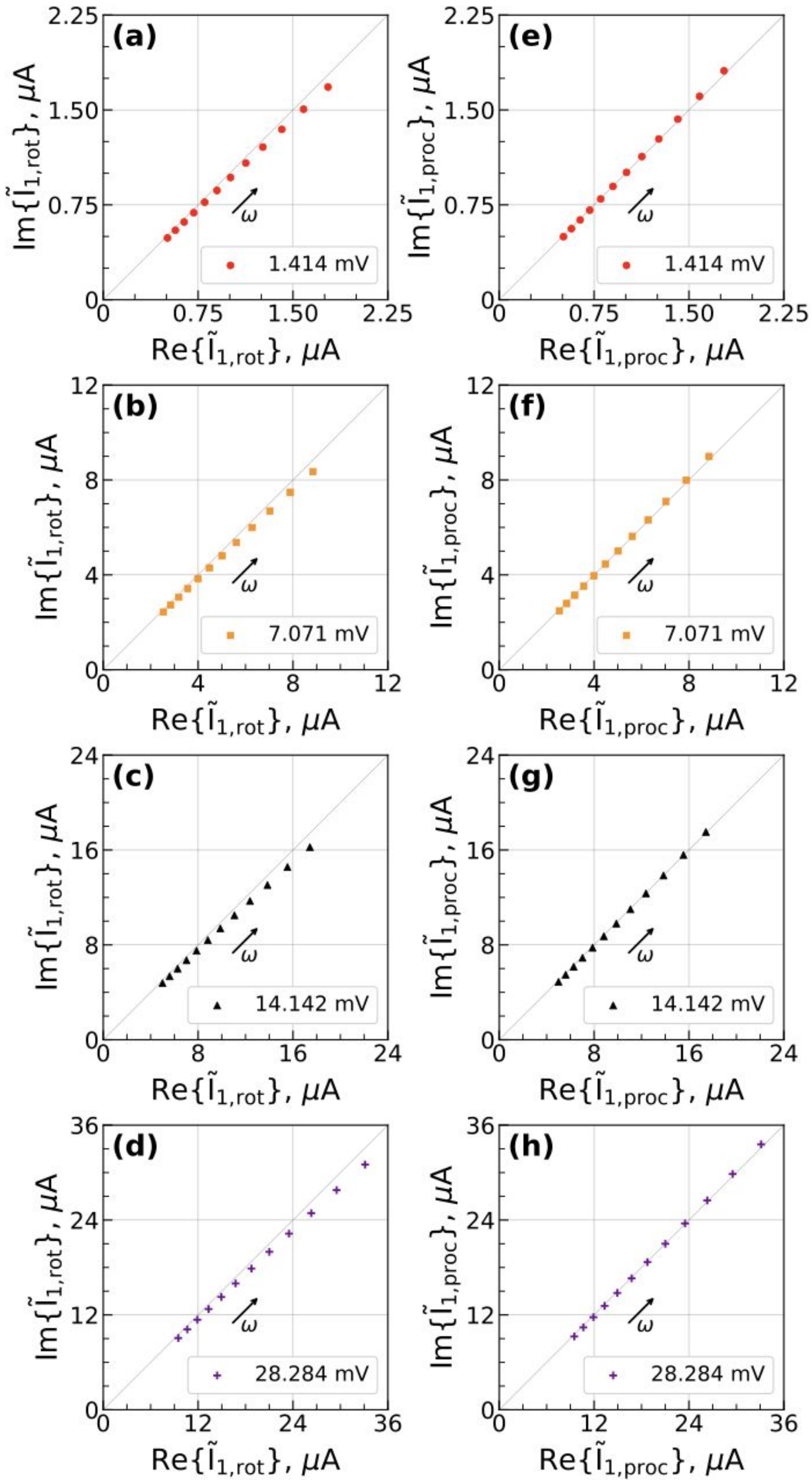

Figure S1 | Comparison of Rotated and Processed First Harmonic Currents. Subplots a-d and e-h depict the rotated $\tilde{I}_{1,rot}$ and processed $\tilde{I}_{1,proc}$ output currents (μA), respectively, for 1.414 mV, 7.071 mV, 14.142 mV, and 28.284 mV potential modulations. The processed currents are corrected by $C_{dl,exp}$= 1.21 $\times$ 10^{-5} Farad. The 45˚ lines represent ideal, semi-infinite Warburg behavior, with frequency increasing in the direction of the overlayed arrows. Replicates are omitted for clarity.

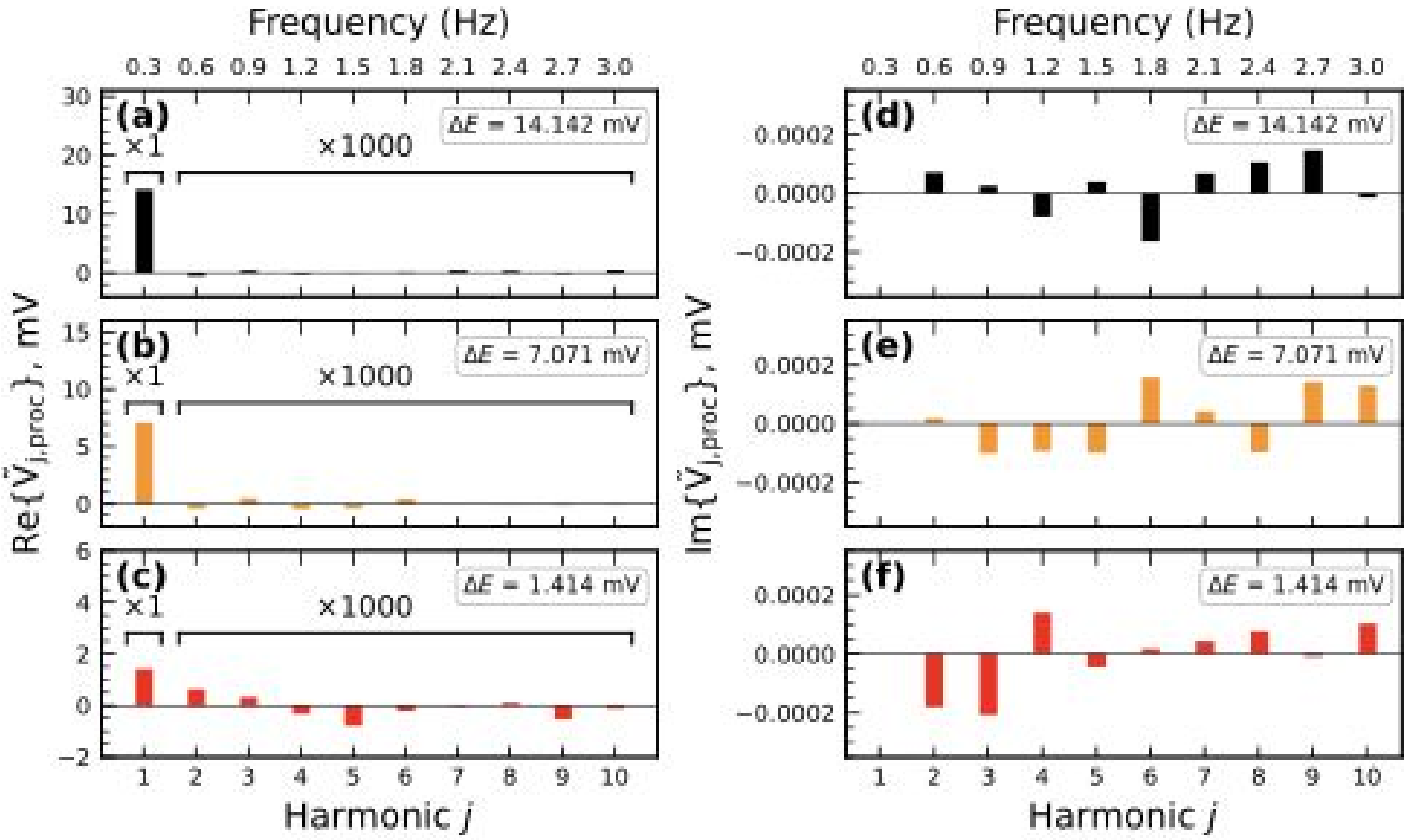

Figure S2 | Experimental Potential Harmonics ($\tilde{V}_{j,proc}$) at Fundamental Frequency of 0.3 Hz. Subplots a-c and d-f depict the real and imaginary processed current output (μA), respectively, for the first ten harmonics ($j = 1$ to 10) generated by a 14.142 mV (a,d), 7.071 mV (b,e) and 1.414 mV (c,f) peak single-sine potential modulations at 0.3 Hz. The higher harmonic ($j \geq 2$) signals are magnified by the ' × ' times label above each bar to improve resolution against the y-axis.

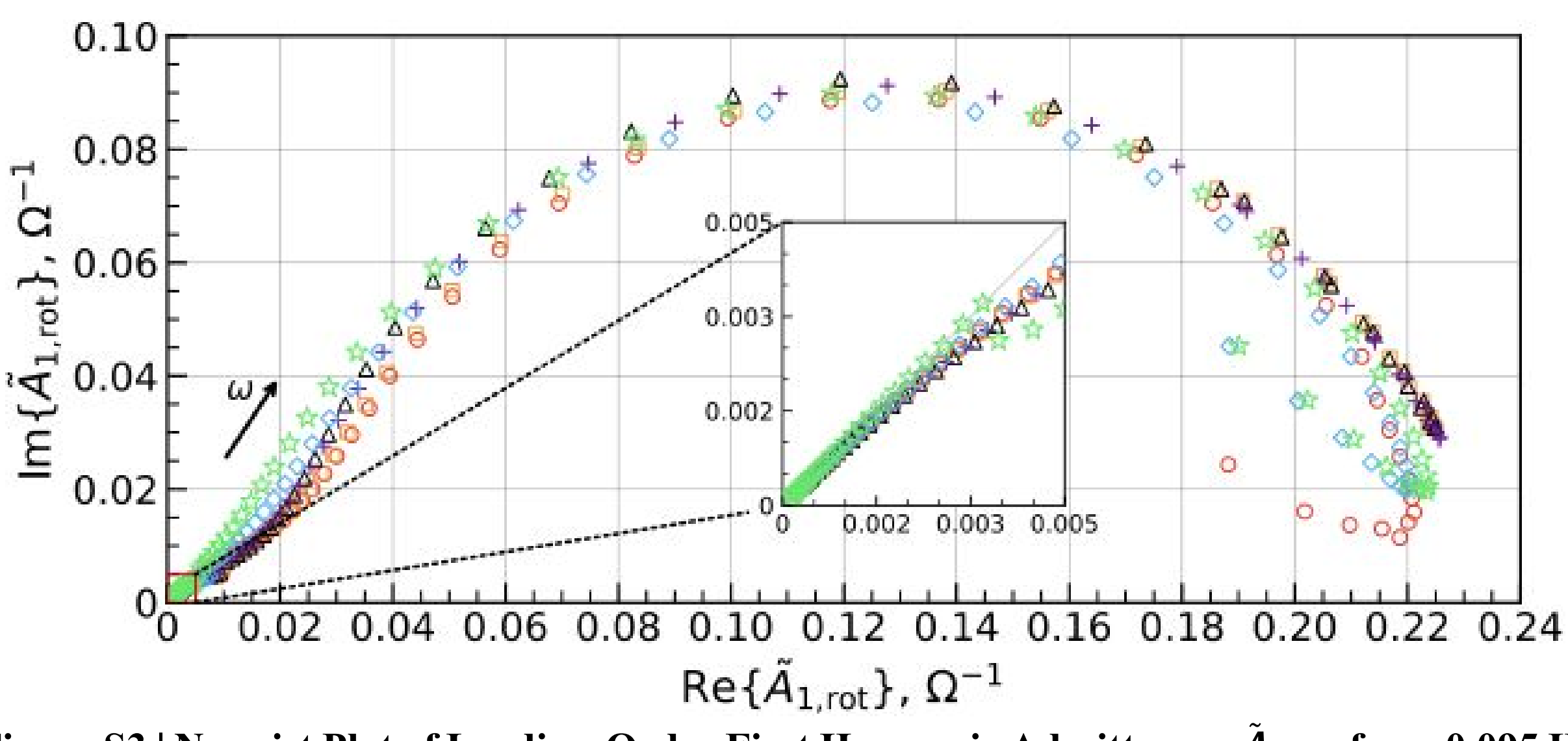

Figure S3 | Nyquist Plot of Leading-Order First Harmonic Admittances $\tilde{A}_{1,rot}$ from 0.095 Hz to 1.5 MHz. Potential modulations (ΔE) of 1.414 mV, 7.071 mV, 14.142 mV, 28.284 mV, 70.711 mV, and 141.421 mV are indicated by red circles, orange squares, black triangles, purple crosses, blue diamonds, and green X's, respectively. Frequency increases in the direction of the overlayed arrow. A plot zoomed in near the origin is overlayed at the center of the Figure, emphasizing the diffusion-limited 45° behavior we analyzed. Replicates are omitted for clarity.

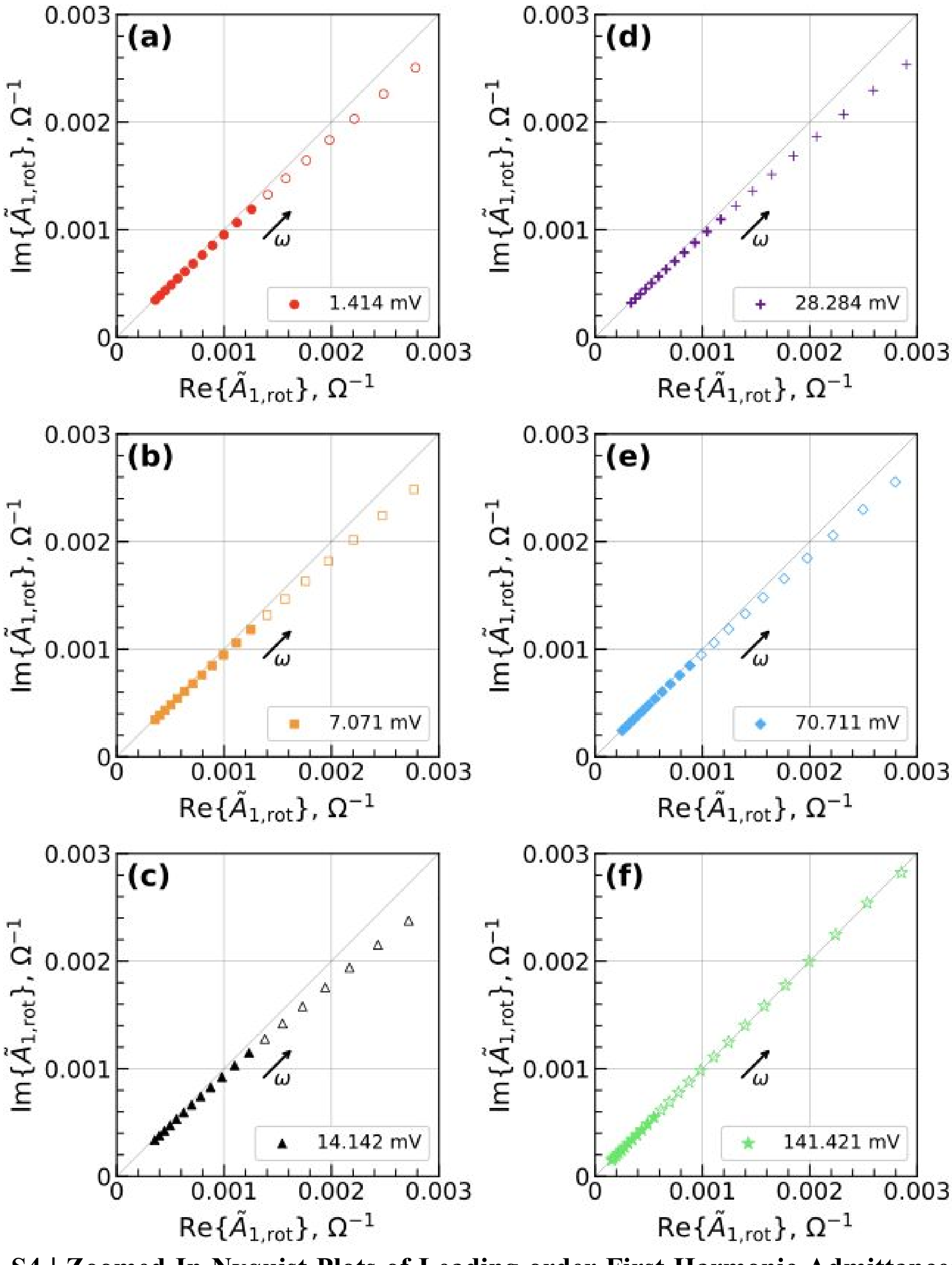

Figure S4 | Zoomed-In Nyquist Plots of Leading-order First Harmonic Admittances $\tilde{A}_{1,rot}$ Near Origin. Subplots (a)-(f) correspond to the rotated first harmonic admittances measured at potential modulations (ΔE) of 1.414 mV, 7.071 mV, 14.142 mV, 28.284 mV, 70.711 mV, and 141.421 mV, respectively. The 12 smallest frequencies (< 1.2 Hz) for Run 1 are indicated by filled in markers. The 45˚ lines represent ideal, semi-infinite Warburg behavior, with frequency increasing in the direction of the overlayed arrows. Replicates are omitted for clarity. Demonstrates that the selected 12 frequencies remain on the 45˚ line for potential modulations ≤ 28.284 mV, where the influence of interfacial capacitance or charge transfer kinetics on the diffusion-limited rates is minimized.

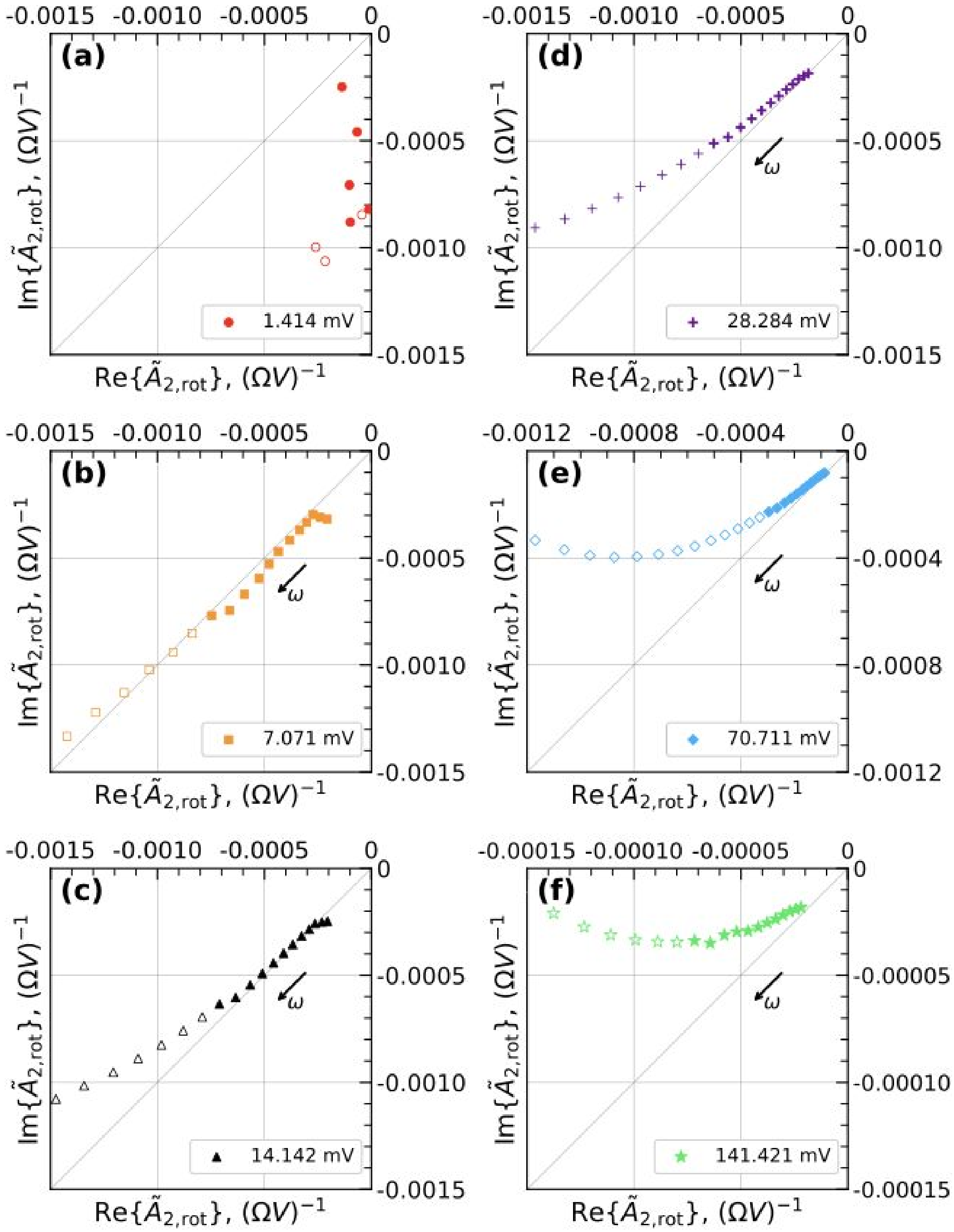

Figure S5 | Zoomed-In Nyquist Plots of Leading-order Second Harmonic Admittances $\tilde{A}_{2,rot}$ Near Origin. Subplots (a)-(f) correspond to the rotated second harmonic admittances measured at potential modulations (ΔE) of 1.414 mV, 7.071 mV, 14.142 mV, 28.284 mV, 70.711 mV, and 141.421 mV, respectively. The 12 smallest frequencies (< 1.2 Hz) for Run 1 are indicated by filled in markers. The 45˚ lines represent ideal, semi-infinite Warburg behavior, with frequency increasing in the direction of the overlayed arrows. Replicates are omitted for clarity. Demonstrates that the selected 12 frequencies remain on the 45˚ line for potential modulations ≤ 28.284 mV, where the influence of interfacial capacitance or charge transfer kinetics on the diffusion-limited rates is minimized.

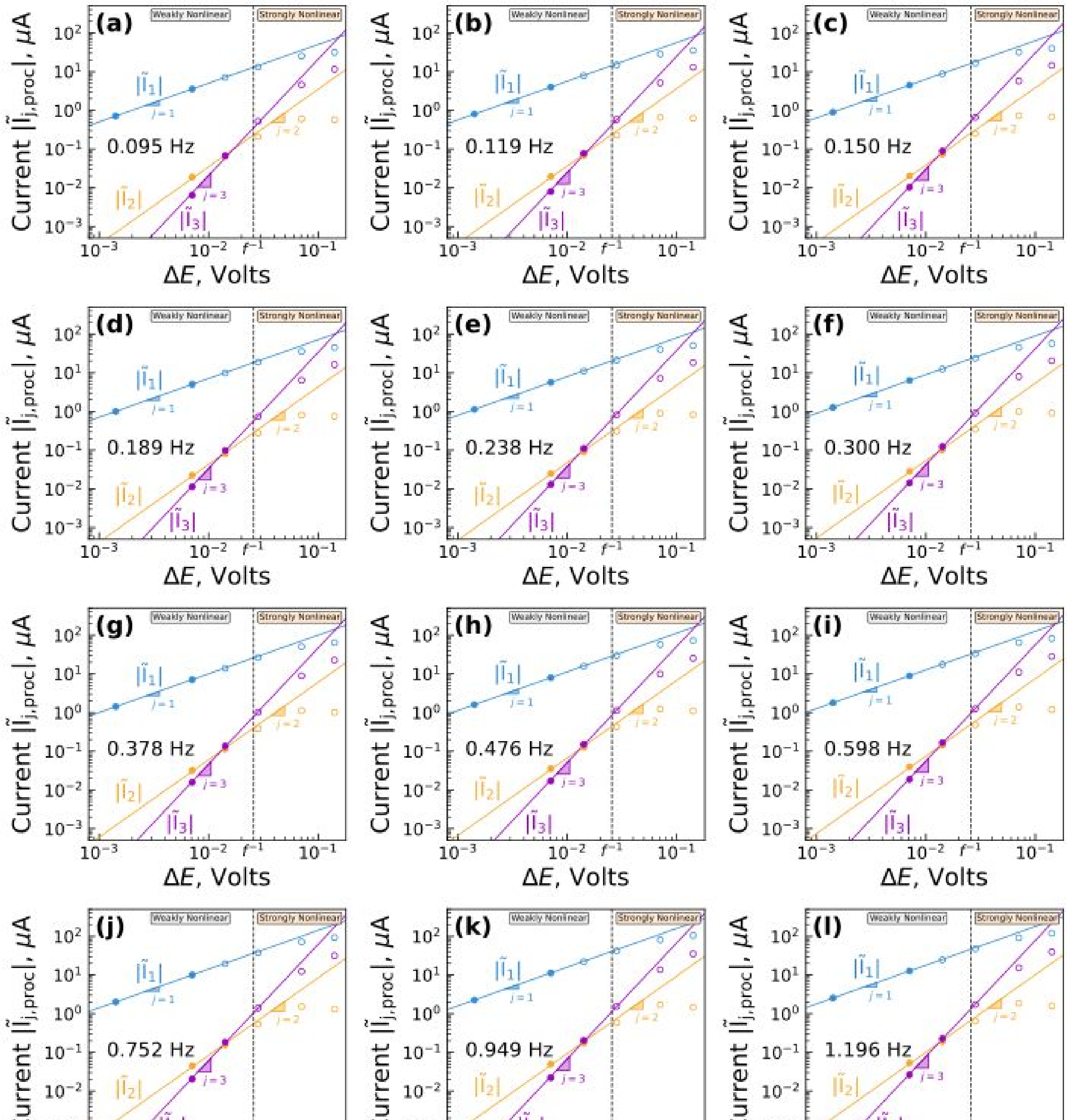

Figure S6 | Run 1 Replicate Data for Experimental Leading-Order Models of Current Magnitudes $\left|\tilde{I}_j\right|$ Versus Potential Modulation (ΔE) at 12 Smallest Frequencies. Same analysis as in Figure 3 of the main text, shown here in subplots (a)-(l) for Run 1 experimental replicates from frequencies 0.095 to 1.196 Hz, labeled respectively.

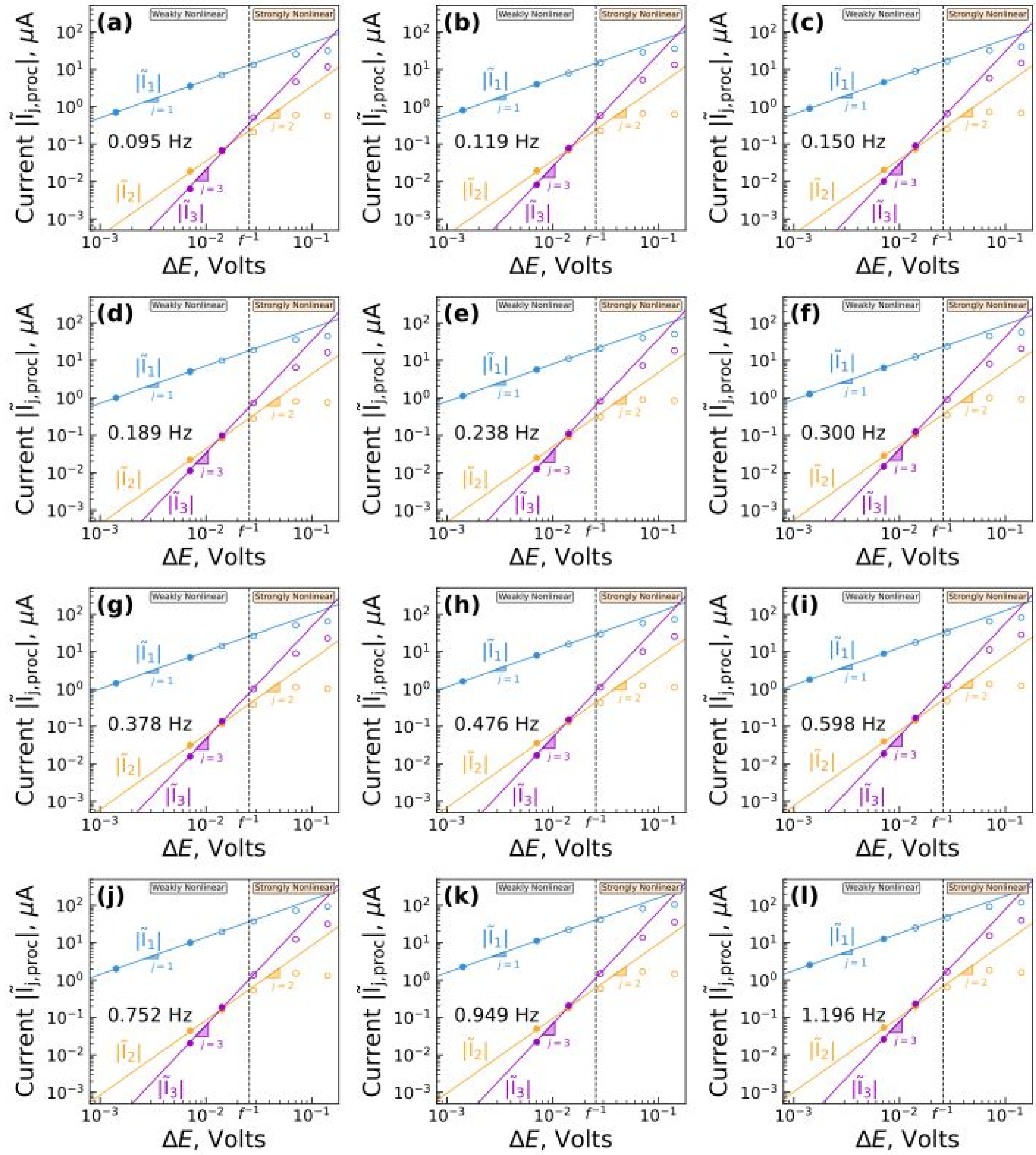

Figure S7 | Run 2 Replicate Data for Experimental Leading-Order Models of Current Magnitudes $\left|\tilde{I}_j\right|$ Versus Potential Modulation (ΔE) at 12 Smallest Frequencies. Same analysis as in Figure 3 of the main text, shown here in subplots (a)-(l) for Run 2 experimental replicates from frequencies 0.095 to 1.196 Hz, labeled respectively.

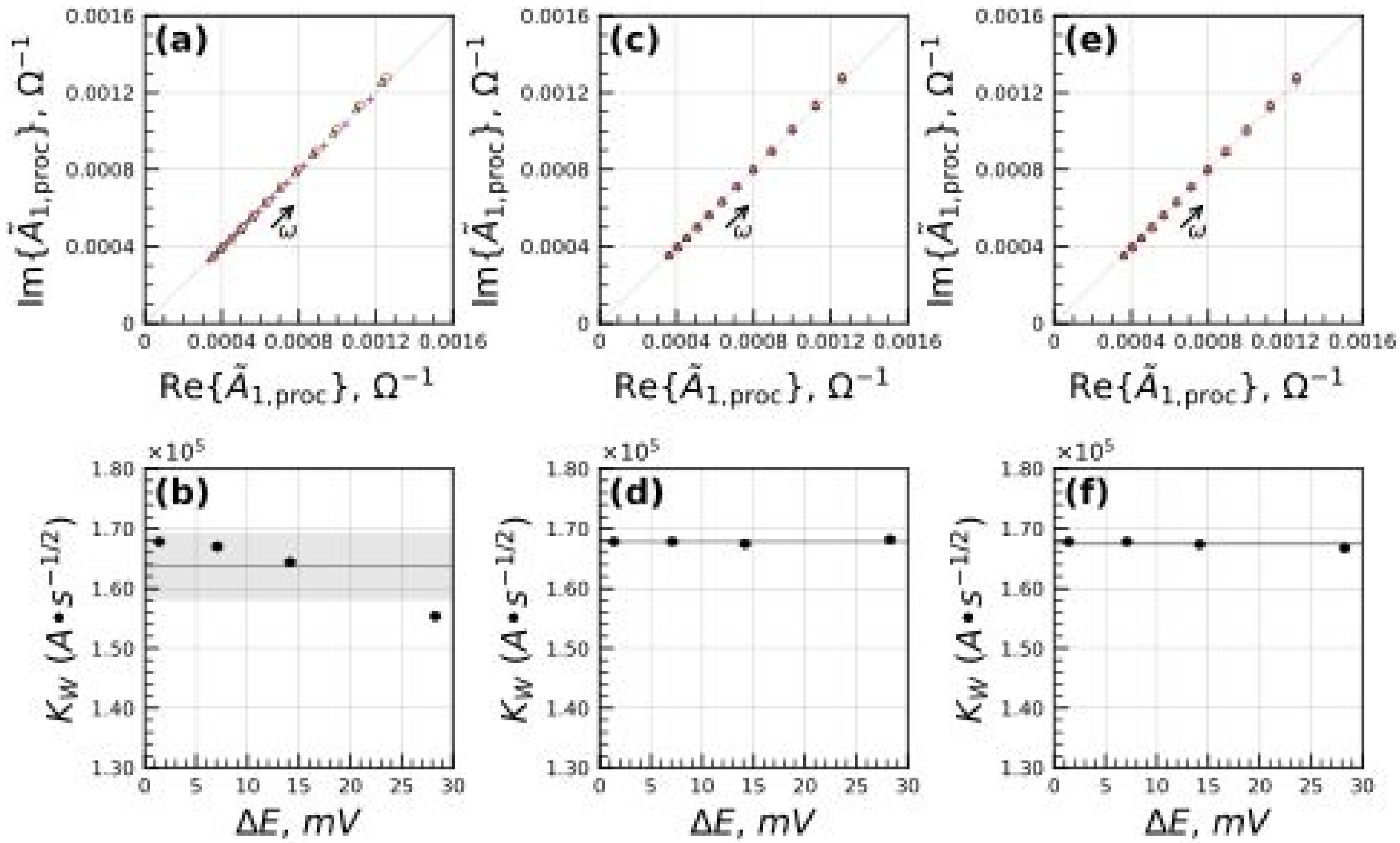

Figure S8 | Replicate Data for Nonlinear Corrections on the Experimental First Harmonic Warburg Admittance $\tilde{A}_1$ and Fitted Parameter K_W. Potential modulations (ΔE) of 1.414 mV, 7.071 mV, 14.142 mV, and 28.284 mV are indicated by red circles, orange squares, black triangles, and purple crosses, respectively. Same analysis as in Figure 5 of the main text, shown here for Run 2 experimental replicates of the (a) leading-, (c) 4th-, and (e) 6th-order corrected first harmonic admittances. Corresponding K_W parameter fits are shown in (b), (d), and (f). Replicate data was omitted from Figure 5 for clarity; however, all experimental replicates were included in K_W parameter fitting, where panels (b), (d), and (f) are identical to data found in main text.

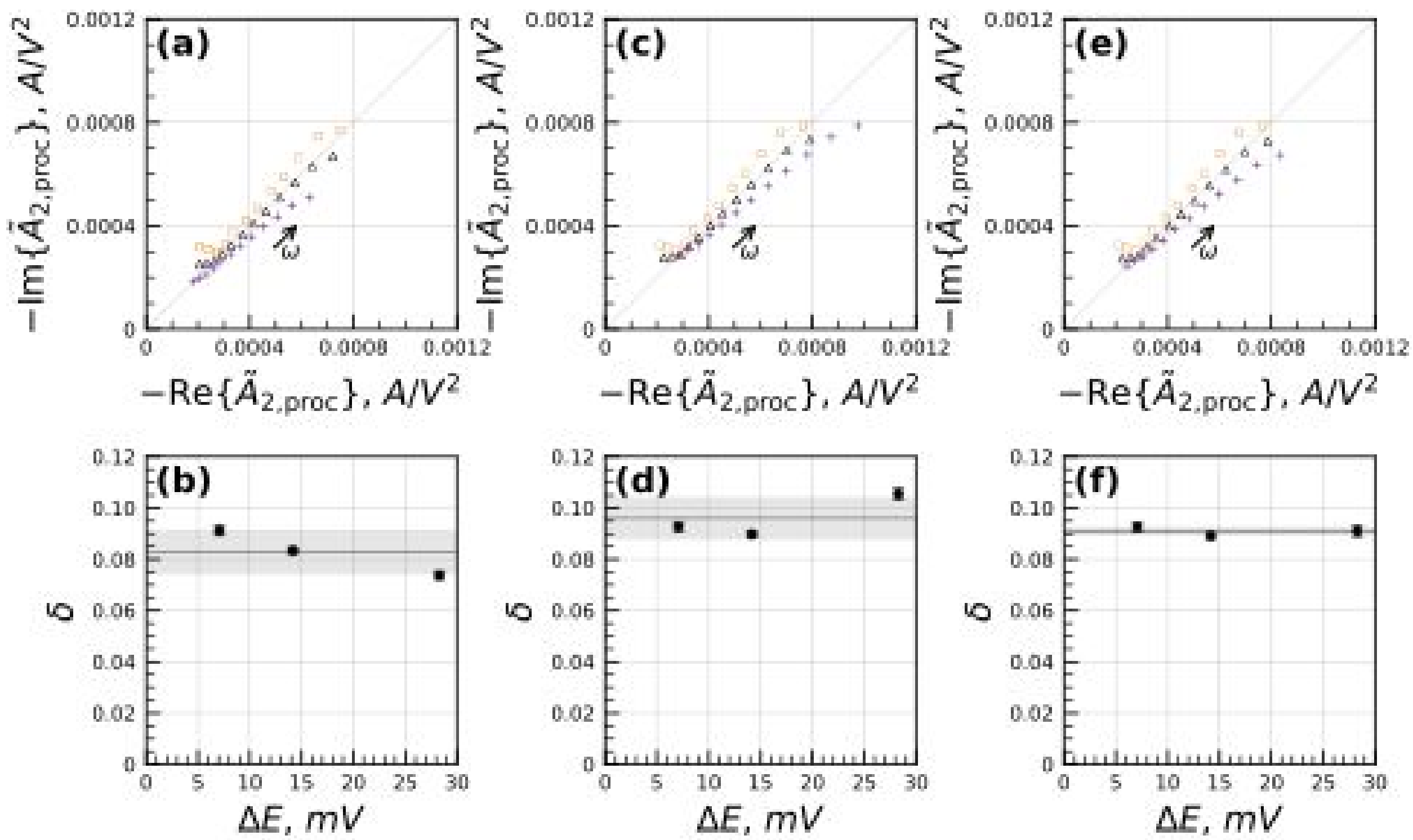

Figure S9 | Replicate Data for Impact of Nonlinear Corrections on the Experimental Second Harmonic Warburg Admittance $\tilde{A}_2$ and Fitted Parameter $\boldsymbol{\delta}$. Potential modulations (ΔE) of 7.071 mV, 14.142 mV, and 28.284 mV are indicated by orange squares, black triangles, and purple crosses, respectively. Same analysis as in Figure 6 of the main text, shown here for Run 2 experimental replicates of the (a) leading-, (c) 4th-, and (e) 6th-order corrected second harmonic admittances. Corresponding δ parameter fits are shown in (b), (d), and (f). Replicate data was omitted from Figure 6 for clarity; however, all experimental replicates were included in δ parameter fitting, where panels (b), (d), and (f) are identical to data found in main text.
